# Multi Omics Analysis Reveals That Compound Radix Pulsatillae and Lactic Acid Bacteria Reprogram the Microbiome Metabolome Network in *Oat* Silage

**DOI:** 10.3390/ijms27125577

**Published:** 2026-06-20

**Authors:** Yuanyuan Jing, Haoran Wang, Heng Jiang, Hui Qu, Guolin Yang, Zhennan He, Siyi Wang, Bin Liu, Fengqin Gao

**Affiliations:** 1 Institute of Grassland Research, Chinese Academy of Agricultural Sciences, No. 120, East Ulanqab Street, Saihan District, Hohhot 010010, China; jingyuanyuan@caas.cn (Y.J.); 18864833878@163.com (H.W.); jiangheng@tju.edu.cn (H.J.); quhui@caas.cn (H.Q.); 16634230300@163.com (G.Y.); a2964898754@163.com (Z.H.); 17860788357@163.com (S.W.); 2 Institute of Animal Husbandry, Inner Mongolia Academy of Agricultural & Animal Husbandry Sciences, No. 22 Zhaojun Road, Yuquan District, Hohhot 010031, China

**Keywords:** Compound Radix Pulsatillae, lactic acid bacteria, *oat* silage, multi-omics, microbiome, metabolome

## Abstract

*Oat* (*Avena sativa* L.) silage fermentation often fails due to insufficient lactic acid bacteria (LAB) and low water-soluble carbohydrate content. We investigated the effects of Compound Radix Pulsatillae (CRP; 40 g/kg FM) alone or combined with a commercial LAB inoculant (containing *L. plantarum*, *L. buchneri*, and *Enterococcus faecium*, CRP_LA) on *oat* silage after 60 days. Compared to control (CK), both CRP and CRP_LA increased dry matter and water-soluble carbohydrate retention while reducing fiber components and ammonia nitrogen (*p* < 0.05). CRP_LA exhibited superior fermentation quality (lowest pH 4.82, highest lactic acid 47.83 g/kg DM). Using 16S rRNA sequencing and UPLC-MS/MS metabolomics integrated with weighted gene co-expression network analysis (WGCNA), we identified a brown module strongly associated with CRP_LA treatment. Six hub metabolites, belonging to flavonoids, terpenoids, alkaloids, phenolic acids, and nucleotide derivatives, were significantly elevated in CRP_LA silage and showed strong correlations with *Lactobacillus* abundance and fermentation quality parameters. Correlation-based network analysis revealed that these hub metabolites positively correlated with Lactobacillus abundance, lactic acid, and water-soluble carbohydrate retention, while negatively correlating with spoilage microorganisms (*Enterobacter*, *Acinetobacter*, *Leuconostoc*) and ammonia nitrogen. This multi-omics study provides a metabolite-centric molecular map of the silage microecosystem reshaped by CRP and LAB co-fermentation. The identified hub metabolites—with predicted antimicrobial, antioxidant, and plant-protective functions—represent potential quality markers for functional silage additive development. Mechanistic validation via targeted metabolite supplementation or pathway-specific gene expression analysis is warranted in future studies.

## 1. Introduction

High-quality forage is the basic guarantee for producing premium animal products. Ensiling technology can effectively preserve forage nutrients and reduce losses, and it is currently the most widely used forage processing method [1]. The essence of ensiling lies in a microbial-driven process: water-soluble carbohydrates (WSCs) are fermented into organic acids, principally lactic acid, which rapidly lowers pH and suppresses spoilage microorganisms. Yet many forages fail to ferment properly due to complex epiphytic microbiota or insufficient WSC, leading to nutrient loss and accumulation of undesirable fermentation products. This not only wastes resources but also threatens livestock health. *Oat* (*Avena sativa* L.) is a high-quality forage widely used for ruminants worldwide. However, *oat* silage is notoriously prone to failure because of low WSC content and scarce epiphytic lactic acid bacteria (LAB). The consequences include severe dry matter (DM) loss, butyric acid accumulation, and excessive proteolysis [2,3]. At the molecular level, these defects reflect disrupted microbial metabolic pathways—for instance, amino acid fermentation taking the place of glycolysis—and inactivation of functional microbial populations. Therefore, developing silage additives that can steer the microbial metabolic network in a desired direction is a key strategy to improve *oat* silage quality.

Research over the years has shown that silage quality can be improved by adding chemical preservatives (e.g., formic acid, acetic acid), exogenous microorganisms (mainly LAB), or plant extracts. LAB are widely used because of their central role in ensiling, but single-strain inoculants, although they accelerate lactic acid accumulation (pH can drop to around 4.2), often fail to completely control harmful microbes. The result is elevated ammonia nitrogen, increased nutrient losses, and compromised aerobic stability, which leads to secondary spoilage after silo opening [4]. Chemical additives like formic or propionic acid do inhibit spoilage organisms, but at high concentrations they impart pungent odors and bitter tastes, reducing palatability [5,6]. More recently, fermentation of Chinese herbal medicines (CHMs) has drawn interest because it can generate new bioactive metabolites through microbial transformation. CHM fermentation is known to enhance pharmacological activity, reduce toxicity, and produce novel bioactive compounds [7]. Evidence suggests that CHM components often require modification by microbial enzymes during fermentation to become bioactive [7,8]. This implies that in the complex fermentation ecosystem of silage, CHM ingredients and microorganisms may interact at the molecular level, potentially reshaping the fermentation micro-environment by modulating specific metabolic pathways or signaling systems.

Compound Radix Pulsatillae (CRP) is a classic formula from Zhang Zhongjing’s Treatise on Febrile Diseases. It consists of *Pulsatilla chinensis*, *Coptis chinensis*, *Phellodendron amurense*, and *Fraxinus chinensis*, and is traditionally used for clearing heat, detoxifying, cooling blood, and stopping diarrhea. Its major active constituents include esculin, fraxin, esculetin, fraxetin, columbamine, coptisine, palmatine chloride, and berberine hydrochloride [9]. These compounds have been shown to possess broad-spectrum antibacterial, anti-inflammatory, and antioxidant activities [10,11,12,13]. CRP exerts its antibacterial effects through molecular mechanisms such as inhibiting bacterial quorum sensing and disrupting cell membrane integrity. Studies have also reported that CRP powder inhibits biofilm formation of *Salmonella* pullorum [14] and *Salmonella* typhimurium [15], thereby preventing infection. When used as a silage additive, CRP suppresses various harmful bacteria and promotes LAB growth [16], making it a promising green silage additive. Nevertheless, systematic research on the combined application of CRP and LAB in silage, especially regarding microbial community structure and metabolite profiles is still lacking.

Against this background, we propose the following hypothesis: the combined use of CRP and LAB synergistically regulates the microbial metabolic network and induces the accumulation of specific functional metabolites, thereby improving *oat* silage quality. In this study, we for the first time combined CRP with a compound LAB inoculant as a silage additive for *oat*. We evaluated its effects on silage quality and, more importantly, systematically analyzed how CRP alone and CRP plus LAB reshape the microbial community and metabolome of *oat* silage. Our aim was to unravel, at the molecular level, the metabolic network mechanisms underlying this additive effect potential and to provide a theoretical basis for the rational design of functional silage additives.

## 2. Results

### 2.1. Oat Silage Quality

Table 1 summarizes the effects of Compound Radix Pulsatillae (CRP) and lactic acid bacteria (LAB) on the fermentation and nutritional quality of *oat* silage. The initial chemical composition of fresh oat is shown in Table 2. Regarding fermentation parameters, both CRP and CRP_LA treatments significantly lowered pH compared to the control (CK) (*p* < 0.05). Among them, the CRP_LA group achieved the lowest pH (4.82) and the highest lactic acid content (LA) (47.83 g/kg), which was significantly greater than that of CRP (36.50 g/kg) and CK (24.22 g/kg) (*p* < 0.001). In contrast, acetic acid (AA) in CRP_LA (12.61 g/kg) was markedly lower than in CK and CRP (*p* < 0.01), and propionic acid (PA) in both additive treatments was significantly lower than in CK (*p* < 0.01). Butyric acid (BA) was not detected in any group. For nutritional quality, CRP_LA had significantly higher dry matter (DM) (36.74%) and water-soluble carbohydrate (WSC) (37.70 g/kg) than the other two groups (*p* < 0.001), and the lowest neutral detergent fiber (NDF) (512.32 g/kg) and acid detergent fiber (ADF) (309.84 g/kg). Crude protein (CP) content in the additive treatments was slightly lower than in CK (*p* < 0.001); given that CRP_LA also had the highest DM, this difference may be partly due to a concentration effect. Moreover, the ammonia nitrogen/total nitrogen ratio (AN/TN) was lowest in CRP_LA (3.79%), significantly lower than in CRP (4.59%) and CK (7.01%) (*p* < 0.001), indicating that the combination of CRP and LAB effectively limits excessive proteolysis.

Overall, adding CRP alone effectively lowered pH and inhibited the conversion of available CP to AN, thereby improving fermentation quality. Furthermore, when combined with lactic acid bacteria, it further promoted lactic acid fermentation, enhanced the retention of dry matter and water-soluble carbohydrates, reduced fiber content, and more significantly suppressed ammonia nitrogen formation, exhibiting a more pronounced synergistic effect. Thus, the combined use of CRP and LAB led to a more comprehensive improvement in both fermentation and nutritional quality of *oat* silage.

### 2.2. Microbial Diversity of Fresh Oat and Oat Silage

**Figure 1:**
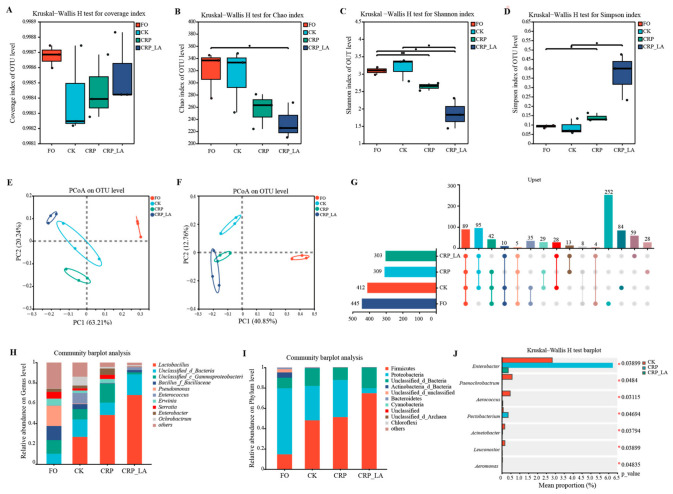
*Oat* raw materials and *oat* silage microorganisms diversity and microbial composition. (**A**) Coverage index. (**B**) Chao1 index. (**C**) Shannon index. (**D**) Simpson index. (**E**) PCoA based on unweighted UniFrac distance. (**F**) PCoA based on weighted UniFrac distance. (**G**) Upset plot of OTUs. (**H**) Relative abundance at the genus level. (**I**) Relative abundance at the phylum level. (**J**) Kruskal–Wallis H test of significant genus. FO: fresh *oat*s.

The microbial diversity of fresh *oat* (FO) and silage samples from different treatment groups was analyzed by high-throughput sequencing, and the results are shown in Figure 1. The Coverage index for all groups exceeded 0.99 (Figure 1A), indicating that sequencing depth was sufficient to cover the vast majority of microorganisms in the samples. Compared with FO, the Chao index decreased significantly after ensiling (Figure 1B), while the Shannon index decreased and the Simpson index increased (Figure 1C,D). This suggests that the ensiling process markedly reduced microbial richness and diversity, while promoting the growth of dominant bacteria (e.g., lactic acid bacteria, LAB) and increasing their relative abundance in the community. This effect was particularly pronounced in the CRP_LA group. Principal coordinate analysis (PCoA) (Figure 1E,F) showed that the microbial community structure of all silage groups was clearly separated from that of FO, and there were also significant differences between the additive-treated groups (CRP and CRP_LA) and the control (CK).

The Upset plot drawn at the OTU level (Figure 1G) revealed that the CRP and CRP_LA groups contained fewer unique microorganisms, indicating that the additives substantially reshaped the microbial community composition. At the phylum level (Figure 1I), FO was dominated by Proteobacteria (65.13%), while Firmicutes accounted for only 14.68%. After 60 days of ensiling, the relative abundance of Firmicutes increased significantly in all treatment groups, reaching 74.52% in the CRP_LA group (*p* < 0.05), whereas the relative abundance of Proteobacteria decreased correspondingly in each group. These results demonstrate that ensiling profoundly altered the epiphytic microbial community structure of *oat*, and the effects of CRP and CRP_LA treatments were particularly strong. At the genus level (Figure 1H), significant differences in microbial composition were observed among the treatment groups. *Lactobacillus* was the dominant genus in all silage groups, and its relative abundance in the CRP_LA group was significantly higher than in the CK and CRP groups, indicating that the addition of LAB effectively promoted the colonization of lactic acid bacteria. The Kruskal–Wallis H test revealed significant differences among treatments for several genera (Figure 1J), including *Enterobacter*, *Paenochrobactrum*, *Aerococcus*, *Pectobacterium*, *Acinetobacter*, *Leuconostoc*, and *Aeromonas*. For potential spoilage bacteria, the relative abundances of *Enterobacter*, *Acinetobacter*, and *Aerococcus* in the CRP_LA group were significantly lower than those in the CK and CRP groups, indicating that the CRP_LA treatment more effectively inhibited the proliferation of spoilage microorganisms.

Clearly, CRP_LA treatment effectively enriched *Lactobacillus* while suppressing colonization by multiple spoilage bacteria. This directed modulation of microbial community structure is consistent with the quality traits observed in the CRP_LA group (highest LA, lowest pH, lowest AN/TN ratio, and highest DM retention in Table 1), providing a microbiological basis for further understanding the mechanism of action of the additives.

### 2.3. Fermentation Metabolites of Oat Silage

**Figure 2:**
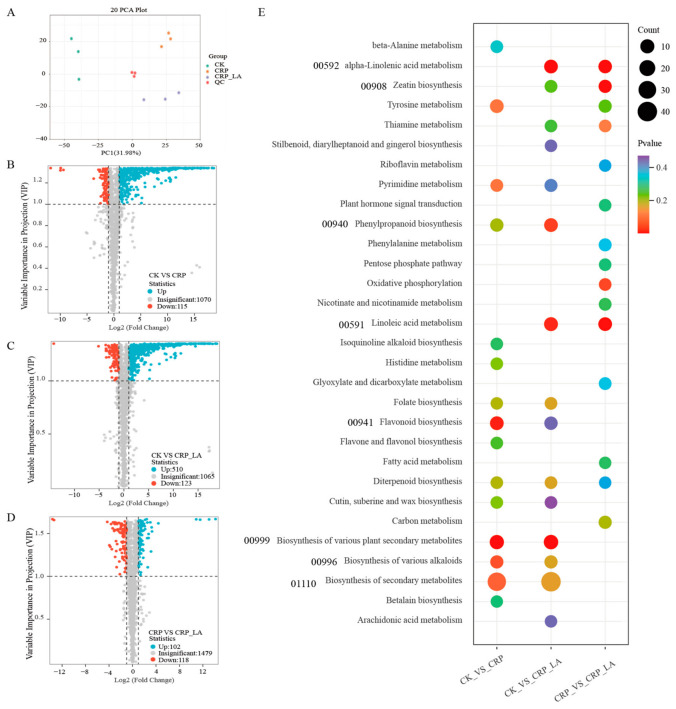
Metabolomics analysis of *oat* silage. (**A**) Principal component analysis (PCA) score plot of all samples. PC1 and PC2 explain 41.27% and 16.85% of the total variance, respectively. Each point represents an individual sample, and samples from the same group are shown in the same color. (**B**–**D**) Volcano plots of differential metabolites between pairwise comparisons. Each point represents one metabolite: green, significantly downregulated; red, significantly upregulated; gray, not significantly different. The x-axis shows the log_2_ fold change (log_2_ FC) of metabolite abundance between the two groups; larger absolute values indicate greater differences. The y-axis shows the variable importance in projection (VIP) value; larger VIP values indicate more reliable differential metabolites. (**E**) KEGG enrichment pathway plot. The size of each dot represents the number of differential metabolites enriched in that pathway (larger dot means more metabolites). The color of the dot represents the significance of enrichment (darker red indicates greater significance).

Principal component analysis (PCA) was performed on the metabolites detected in each group (Figure 2A). The first principal component (PC1) explained 41.27% of the data variance, and the second (PC2) explained 16.85%. The PCA plot showed tight clustering among biological replicates within each group, but clear separation among different treatment groups, indicating that the addition of CRP and CRP_LA significantly affected the metabolite profiles in *oat* silage. Figure 2B–D display the differential metabolites identified in pairwise comparisons between treatments. Compared with CK, the CRP group had 514 upregulated and 115 downregulated metabolites, with 1070 showing no significant difference. In the CRP_LA group, 510 were upregulated and 123 downregulated, while 1065 were not significantly different. Direct comparison between CRP and CRP_LA revealed 102 upregulated and 118 downregulated metabolites in CRP_LA relative to CRP, with 1479 unchanged. These results demonstrate that different additive treatments lead to marked differences in metabolite composition in *oat* silage.

KEGG (Kyoto Encyclopedia of Genes and Genomes) functional annotation and enrichment analysis were carried out based on the differential metabolites (Figure 2E). Compared with CK, both CRP and CRP_LA treatments commonly enriched the pathway “Biosynthesis of various plant secondary metabolites”, likely influenced by the unique bioactive compounds present in Compound Radix Pulsatillae. Meanwhile, compared to CRP alone, the CRP_LA treatment showed reduced enrichment of metabolites related to “Flavonoid biosynthesis” and “Arachidonic acid metabolism”, but increased enrichment of “Phenylpropanoid biosynthesis” and “Linoleic acid metabolism”. Relative to both CK and CRP, CRP_LA uniquely enriched metabolites in “Linoleic acid metabolism” and “alpha-Linolenic acid metabolism”, suggesting that the accumulation of metabolites in these two pathways was driven by the addition of LAB. Moreover, compared to CRP, CRP_LA also significantly enriched metabolites in the “Zeatin biosynthesis” pathway.

### 2.4. Weighted Gene Co-Expression Network Analysis (WGCNA) on the Metabolite Dataset

**Figure 3:**
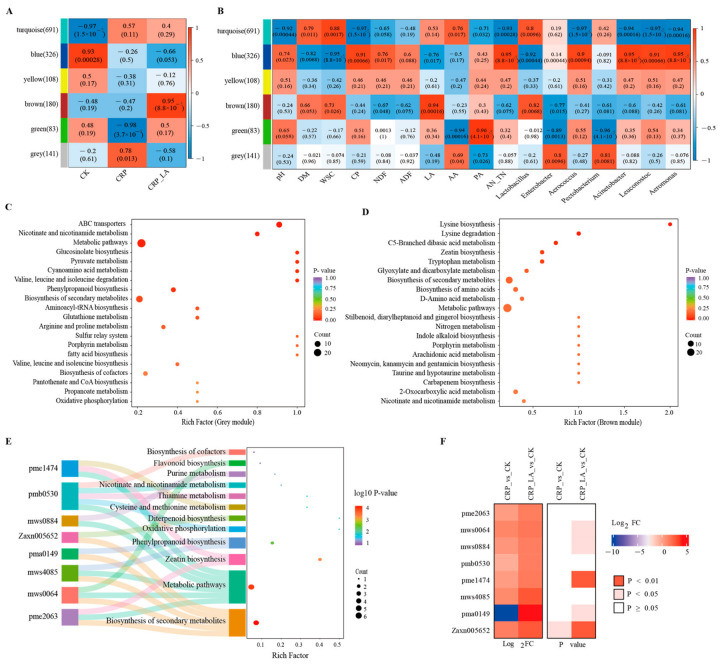
WGCNA-based metabolome analysis. (**A**) Module–group trait relationships. (**B**) Module–trait relationships between each module and silage fermentation parameters. (**C**) KEGG enrichment pathway plot of metabolites in the grey module (associated with CRP treatment). (**D**) KEGG enrichment pathway plot of metabolites in the brown module (associated with CRP_LA treatment). (**E**) KEGG enrichment pathway plot of the eight hub metabolites identified from the intersection analysis. (**F**) Heatmap showing the expression levels (fold change) of the eight hub metabolites across the three treatments.

To further elucidate the regulatory mechanisms underlying additive effects on *oat* silage metabolites, weighted gene co-expression network analysis (WGCNA) was employed to cluster the 1701 detected metabolites into six distinct modules: turquoise, blue, yellow, brown, green, and grey (Figure 3A). The brown module showed a strong positive correlation with the CRP_LA treatment (r = 0.81, *p* < 0.01), whereas the grey module was specifically associated with the CRP treatment (r = 0.67, *p* < 0.05). This finding suggests that CRP and CRP_LA may recruit distinct metabolic modules to reconfigure the silage metabolic network, with the brown module potentially contributing to the superior fermentation phenotype observed in the CRP_LA treatment. Module–trait association analysis (Figure 3B) further indicated significant correlations between individual modules and silage quality parameters (pH, DM, WSC, CP, NDF, ADF, LA, AA, PA, AN/TN) as well as the abundances of key microbial genera (*Lactobacillus*, *Enterobacter*, *Aerococcus*, *Pectobacterium*, *Acinetobacter*, *Leuconostoc*, *Aeromonas*). The CK treatment was characterized by strong enrichment of the blue module, which was rich in free fatty acids, amino acids and derivatives, alkaloids, and phenolic acids. Correlation analysis (Figure 3B) showed that the blue module was positively correlated with pH, CP, NDF, ADF, and AN/TN, and negatively correlated with DM, WSC, LA, and *Lactobacillus* abundance. Moreover, the blue module was positively associated with spoilage genera (*Aerococcus*, *Acinetobacter*, *Leuconostoc*, *Aeromonas*). The blue module was highly enriched in CK but much less abundant in CRP and CRP_LA (Figure 3A), suggesting that the CK silage promoted more spoilage bacteria, thereby generating AN, while also enhancing DM and WSC losses, and inhibiting *Lactobacillus* colonization and LA production.

The CRP treatment significantly upregulated the grey module, which contained a smaller number of metabolites, mainly low-molecular-weight phenolic acids, alkaloids, and some free fatty acids. The grey module was positively correlated with AA and the spoilage genera *Enterobacter* and *Pectobacterium*, and negatively correlated with DM and WSC. No grey module metabolites were detected in CRP_LA or CK. Meanwhile, CRP strongly down-regulated the green module, which was also suppressed in CK and CRP_LA, and this module was negatively correlated with PA production. These observations indicate that CRP contains unique bioactive compounds that probably inhibited PA synthesis while yielding higher AA concentrations. The CRP_LA treatment was predominantly associated with the brown module. Its metabolite composition was dominated by hydroxy fatty acids, lysophosphatidylcholine, phenolamides, and triterpenoid saponins (Supplementary Table S1). These metabolites were positively correlated with WSC retention, LA production, and *Lactobacillus* enrichment, and negatively correlated with pH, AN/TN, and all detected spoilage genera (*Enterobacter*, *Pectobacterium*, *Acinetobacter*, *Leuconostoc*, *Aeromonas*). The brown module was specifically enriched in CRP_LA, and its metabolite levels differed significantly among treatments. This enrichment likely reflects the enhancing effect of added LAB, as brown module metabolites increased only in the presence of LAB (CRP_LA). Compared with CK, both CRP and CRP_LA increased the turquoise module, which contained numerous lignans, coumarins, phenolic acids, and triterpenoids. This module was negatively correlated with pH, AN/TN, and all spoilage bacteria, but also negatively correlated with *Lactobacillus* abundance. The turquoise module was enriched in CK and CRP, but showed moderate abundance in CRP_LA (Figure 3A). Collectively, these results demonstrate that different additive treatments significantly affect silage fermentation quality and microbial community structure by modulating the abundance of specific metabolite modules, particularly the brown and blue modules.

To precisely identify metabolites that play core functional roles under each treatment, we performed an intersection analysis between brown module metabolites (associated with CRP_LA) and differential metabolites from the CK vs. CRP_LA comparison (Figure 3C), and similarly between grey module metabolites (associated with CRP) and differential metabolites from CK vs. CRP_LA (Figure 3D). Enrichment analysis of the resulting metabolites (Figure 3E) identified eight key metabolites, which were primarily enriched in the pathways “Metabolic pathways” (ko01100) and “Biosynthesis of secondary metabolites” (ko01110). KEGG enrichment further confirmed that these eight metabolites play central regulatory roles in the CRP_LA treatment (Figure 3E,F; Table 3). They include a plant hormone (cis-zeatin), a flavonoid (eriodictyol), nucleotide derivatives (cyclic 3′,5′-adenylic acid, 5′-deoxy-5′-(methylthio)adenosine), a phenolic ester (sinapoyl malate), and a terpenoid (pisiferic acid). Heatmap analysis showed that all of these metabolites were significantly upregulated in the CRP_LA and CRP treatments, except for pma0149 (sinapoyl malate), which was suppressed by CRP alone but strongly induced when LAB was added.

### 2.5. Correlation Networks of Key Metabolites with Fermentation Quality and Microorganisms

**Figure 4:**
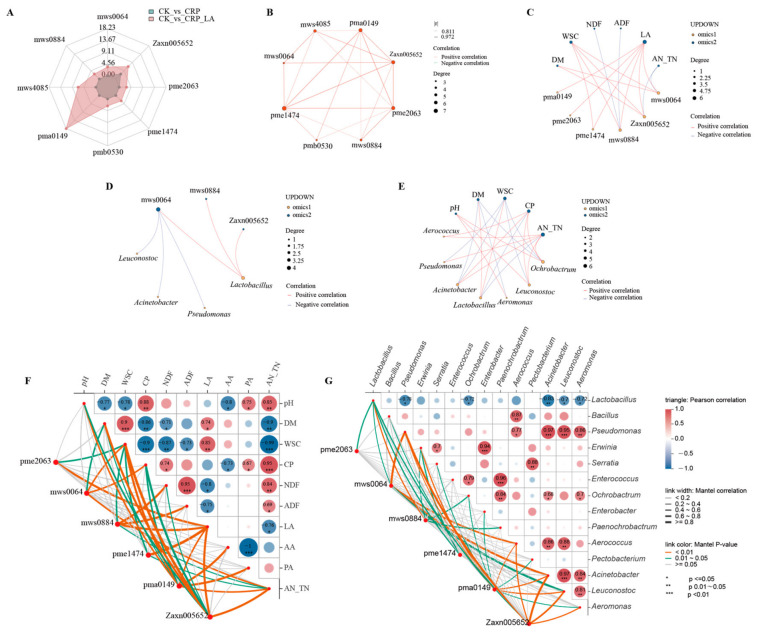
Correlation networks of key metabolites with fermentation quality and microorganisms. (**A**) Radar chart of key metabolites based on fold change (FC) values. (**B**) Correlation network among key metabolites. (**C**) Network correlation between key metabolites and silage nutritional/fermentation quality. (**D**) Network correlation between key metabolites and key differential microorganisms. (**E**) Network correlation between key differential microorganisms and fermentation/nutritional quality. (**F**) Mantel test between key metabolites and fermentation/nutritional quality. (**G**) Mantel test between key metabolites and key differential microorganisms.

To visualize the changes in the eight hub metabolites across treatments, we generated a heatmap of fold-change values (Figure 4A) and a correlation network among the metabolites (Figure 4B). All key metabolites were significantly upregulated under CRP_LA treatment (fold change > 2, *p* < 0.05), with tight positive correlations among them, suggesting that CRP_LA induced a more pronounced metabolic response. Figure 4C,D present metabolite–quality–microbe correlation networks. Mantel analysis revealed that six of the hub metabolites (pme2063, mws0064, mws0884, pme1474, pma0149, Zaxn005652) were positively correlated with LA (Figure 4D,F). Four metabolites (mws0884, pme1474, pma0149, Zaxn005652) were positively correlated with WSC, and two (pma0149, Zaxn005652) with DM (Figure 4C). Three metabolites (mws0064, mws0884, Zaxn005652) were positively correlated with the dominant genus *Lactobacillus* (Figure 4D,G); mws0064 was negatively correlated with the spoilage genera *Leuconostoc*, *Acinetobacter*, and *Pseudomonas* (Figure 4C), and positively correlated with AN production (Figure 4E). These results indicate that both abundant and differential microorganisms, together with the key metabolites, jointly influence the *oat* silage fermentation process and consequently its quality. Based on the expression levels of the hub metabolites and their comparisons among treatments, we summarized their main functions and whether they are induced by CRP or CRP_LA, as shown in Table 3. Some metabolites (e.g., Zaxn005652, mws0064, mws0884) were induced by CRP addition, and their levels were further enhanced by LAB (CRP_LA), indicating that CRP contributes to antioxidant and antimicrobial metabolic functions. Conversely, CRP alone suppressed the production of one metabolite (pma0149). Of these, six key metabolites (pme2063, mws0064, mws0884, pme1474, pma0149, and Zaxn005652), which were significantly differential and critical in the CRP_LA treatment, significantly influenced silage quality and were positively correlated with LA.

## 3. Discussion

### 3.1. Effect of CRP on Oat Silage Quality

The primary goal of ensiling is to create an anaerobic environment that promotes lactic acid bacteria (LAB)-dominated fermentation, lowers pH, and suppresses spoilage organisms and molds, thereby reducing nutrient losses and improving feed stability [4,23,24]. While the absence of a single LAB treatment precludes distinguishing between the independent effect of exogenous LAB and its combined effect with CRP, the present results nevertheless demonstrate that both Compound Radix Pulsatillae (CRP) alone and its combination with LAB (CRP_LA) significantly improved *oat* silage fermentation quality, with CRP_LA being notably more effective. While the optimal pH for well-preserved *oat* silage is generally considered to be 3.8–4.5 [25,26], the CRP_LA treatment in this study achieved the lowest pH (4.82) and the highest lactic acid (LA) content among all treatments (*p* < 0.05), indicating that the exogenous LAB inoculant (Xinlaiwang I, containing *Lactobacillus plantarum* and *L. buchneri*) efficiently utilized WSC to accelerate LA production and acidification, thereby optimizing fermentation quality [24,27,28]. Interestingly, CRP alone, although it did not supply exogenous LAB, still resulted in a significantly lower pH and higher LA content than the control (CK) (*p* < 0.05). This may be attributed to the broad-spectrum inhibitory effects of CRP’s bioactive components (e.g., berberine from Coptis, esculin from Fraxinus) against aerobic microorganisms during the early stage of ensiling [7,29], thus creating a favorable environment for LAB fermentation.

The CRP_LA treatment achieved significantly higher retention of DM, WSC than the other groups. This advantage likely stems from the rapid proliferation of exogenous LAB. These bacteria quickly acidified the environment (pH 4.82), shortened the fermentation lag phase, and suppressed yeasts, molds, and proteolytic bacteria, thereby minimizing WSC consumption and DM loss [28]. Meanwhile, homofermentative lactic acid bacteria convert one molecule of glucose into two molecules of lactic acid, exhibiting high carbon conversion efficiency. In contrast, heterofermentative or contaminant metabolism produces CO_2_ and other gases, resulting in carbon loss. The CRP_LA group was dominated by homofermentation (as evidenced by a high lactic acid/acetic acid ratio), enabling more lactic acid production per unit of WSC consumed while preserving greater residual WSC. Although NDF in the CRP_LA group decreased from 598.64 to 512.32 g/kg DM, absolute values on a fresh weight basis were nearly identical (187.31 vs. 188.23 g/kg), indicating that the apparent reduction was largely a DM concentration artifact rather than genuine fiber degradation. While hemicellulose hydrolysis may release fermentable sugars, it did not produce a net NDF decrease. Fiber data should therefore be evaluated on both fresh weight and DM bases. The absence of BA and the markedly lower AN/TN ratio in CRP_LA further confirm effective inhibition of proteolysis. CRP alone, despite raising LA and lowering pH, showed a significantly higher AA content than CRP_LA, suggesting that without sufficient exogenous LAB, CRP may promote some heterolactic fermentation pathways, diverting part of the WSC to AA instead of LA [30,31]. Notably, although CP content in the CRP group was slightly lower than in CK, its AN/TN ratio was significantly reduced, indicating that proteolysis was indeed suppressed; the small CP decrease may be related to a DM concentration effect. High AA content, while potentially beneficial for aerobic stability after silo opening, also implies lower energy efficiency during fermentation [32]. In summary, CRP alone has the potential to improve *oat* silage fermentation quality by lowering pH and increasing LA, but its effect is weaker than that of CRP combined with LAB. CRP_LA, through rapid acidification by exogenous LAB, significantly reduced losses of DM, WSC, demonstrating superior effects.

### 3.2. Microbial Diversity in Oat Silage

The results of this study showed that the epiphytic microorganisms on fresh *oat* (FO) prior to ensiling were dominated by Proteobacteria (relative abundance > 60%). After 60 days of ensiling, rapid microbial community succession occurred, with the relative abundance of Firmicutes significantly increasing in all treatment groups while Proteobacteria was suppressed. Notably, the abundance of Firmicutes in the CRP_LA group was significantly higher than that in the CRP and CK groups. This shift aligns with the typical pattern of silage fermentation, wherein anaerobic and acidic environments selectively promote the proliferation of facultative/obligate anaerobic Firmicutes while inhibiting aerobic Proteobacteria members—a conclusion consistent with the findings of Yan et al. [33], Zhu et al. [34] and Yang et al. [35]. Firmicutes encompasses the majority of lactic acid bacteria (e.g., *Lactobacillus*), which produce lactic acid through homofermentative pathways and lower pH; higher abundance indicates more complete fermentation [1,4,36]. At the genus level, *Lactobacillus* became the absolute dominant genus after ensiling, with abundance ranking as CRP_LA > CRP > CK. Xin et al. [31]. and Muck et al. [37] reported that *Lactobacillus* proliferation is a hallmark of successful ensiling, as it drives rapid acidification, suppresses clostridia and other spoilage organisms, and ensures silage stability and nutritional quality—consistent with our findings. Both CRP and CRP_LA treatments significantly reduced alpha diversity indices and the number of unique microorganisms, indicating that the additives intensified the acidic environment, selectively promoting acid-tolerant microorganisms (e.g., LAB) while inhibiting pH-sensitive harmful bacteria [37,38,39].

Kruskal–Wallis H test revealed significant differences among treatments for genera including *Enterobacter*, *Paenochrobactrum*, *Aerococcus*, *Pectobacterium*, *Acinetobacter*, *Leuconostoc*, and *Aeromonas*. Most of these belong to *Proteobacteria* or are considered opportunistic pathogens/spoilage bacteria; their reduced abundance in CRP_LA directly reflects the suppressive effect on harmful microorganisms. Among the differentially abundant genera, *Lactobacillus* was the most abundant. CRP addition significantly promoted the proliferation of Enterococcus and Enterobacter, while CRP_LA further introduced exogenous *Lactobacillus plantarum* and *Lactobacillus buchneri*. Muck et al. [31] pointed out that combination inoculants aim to provide the aerobic stability benefits of *L. buchneri* together with the fermentation efficiency and animal productivity benefits of homofermentative LAB, which is consistent with our findings.

The results of this study indicate that CRP, as a silage additive, can significantly promote *Lactobacillus* colonization and lactic acid fermentation, while facilitating the release of bioactive components from CHM, thereby providing a favorable fermentation environment for antimicrobial and antioxidant microorganisms. Regarding these findings, studies by Gao et al. [40] and Wang et al. [41] have demonstrated that active components of CHM may function as prebiotics to promote the proliferation of beneficial microorganisms in the host; meanwhile, extracellular enzymes (e.g., cellulase) produced by probiotics can disrupt plant cell walls, releasing bioactive components in silage and creating a suitable environment for antimicrobial and antioxidant microorganisms. These findings are consistent with the conclusions of this study. The modulation of microbial succession by CRP and CRP_LA operates through two mechanisms: on one hand, CRP’s natural active ingredients (e.g., alkaloids and polyphenols) promote the colonization and metabolic activity of beneficial bacteria like *Lactobacillus*, accelerating pH decline; on the other hand, exogenous LAB compete with spoilage microbes for nutrients and directly inhibit them through bacteriocins. These steps significantly improve fermentation efficiency, as reflected by the higher LA/AA ratio and lower pH in the CRP_LA group. In conclusion, CRP combined with LAB (CRP_LA) optimizes the microbial community structure (enriching *Lactobacillus* and suppressing spoilage bacteria), thereby significantly improving *oat* silage quality and providing a theoretical basis for the development of green additives.

### 3.3. Analysis of Fermentation Metabolites

This study, using UPLC-MS/MS untargeted metabolomics combined with WGCNA, revealed the global remodeling of the metabolite profile in *oat* silage under different additive treatments. PCA showed clear separation of the metabolite profiles of CRP and CRP_LA from CK, as well as differences between CRP and CRP_LA, indicating that both CRP alone and its combination with LAB significantly altered the metabolite composition during ensiling. KEGG enrichment analysis showed that both CRP and CRP_LA commonly enriched the pathway “Biosynthesis of various plant secondary metabolites”, consistent with the fact that CRP itself is rich in diverse phytochemicals (alkaloids, coumarins, saponins, etc.). Notably, CRP_LA uniquely enriched “Linoleic acid metabolism”, “alpha-Linolenic acid metabolism”, and “Zeatin biosynthesis” pathways, whereas CRP alone did not. Jameson [42] and Wang et al. [43] demonstrated that zeatin metabolism reduces reactive oxygen species accumulation, preserves nutritional quality, and significantly delays leaf senescence. These findings support the hypothesis that CRP_LA treatment may similarly maintain silage color, attenuate proteolysis, and enhance nutritional quality. The specific enrichment of these pathways suggests that exogenous lactic acid bacteria not only accelerated acidification, but also potentially activated intrinsic *oat* defense metabolism and senescence regulatory mechanisms via plant–microbe interactions to preserve silage nutritional quality—a phenomenon rarely documented in ensiling research.

WGCNA clustered the 1701 metabolites into six co-expression modules. The brown module was strongly positively correlated with CRP_LA, the blue module was highly enriched in CK, and the grey module was specifically associated with CRP. Blue module metabolites (free fatty acids, amino acid derivatives, indole/quinoline alkaloids) were positively correlated with pH, AN/TN, and spoilage bacterial abundance, and negatively correlated with DM, WSC, and *Lactobacillus* abundance, suggesting that the blue module may represent characteristics of failed or low-quality fermentation. In contrast, brown module metabolites (including hydroxy fatty acids, phenolamides, lysophosphatidylcholine, and triterpenoid saponins) were positively correlated with WSC retention, LA production, and *Lactobacillus* enrichment, and negatively correlated with pH, AN/TN, and all detected spoilage genera. Phenolamides in the brown module (e.g., avenanthramides, N-feruloyltyramine) are defense metabolites specific to Poaceae, with antioxidant and antifungal activities [44]. The specific enrichment of the brown module under CRP_LA treatment suggests that CRP_LA activated the intrinsic defense metabolic network of *oat*, which may account for the protective effect conferred by exogenous lactic acid bacteria. Within the brown module, we further identified six key metabolites with significantly altered abundances (Table 3). Based on their accumulation patterns across treatments, they can be categorized into three types: Type I (cis-zeatin, MTA) were significantly upregulated only in the presence of exogenous LAB (CRP_LA), with no effect from CRP alone, indicating their accumulation depends on LAB metabolic activity; Type II (eriodictyol, pisiferic acid) were already elevated in CRP and further enhanced in CRP_LA, suggesting that CRP can directly or indirectly induce their formation, furthermore, exogenous lactic acid bacteria exhibited a superior additive enhancing effect. Type III (sinapoyl malate) showed a unique reversal pattern—almost completely suppressed by CRP alone but dramatically increased when LAB was added, indicating that LAB can overcome the inhibitory effect of certain components in CRP and greatly promote the accumulation of this compound. Overall, these metabolites belong to different classes (flavonoids, terpenoids, alkaloids, phenolic acids, nucleotide derivatives), with functions involving antioxidant, antibacterial, and regulation of LAB metabolic activity. Their coordinated upregulation collectively constitutes the metabolic basis for the superior fermentation phenotype of CRP_LA silage.

Because we did not perform chemical characterization of CRP or targeted metabolite supplementation experiments, the exact origins of these metabolites (directly from CRP, synthesized by *oat* plants, or transformed by LAB) and their causal relationships with fermentation quality remain to be further validated. Nevertheless, through multi-omics integration, this study provides the first metabolic network map of *oat* silage under fermentation of a Chinese herbal medicine and LAB, offering metabolite-level targets and a theoretical basis for the development of functional silage additives.

### 3.4. Limitations

(1) The most significant limitation of this study is the absence of a LAB-only treatment group. Consequently, we were unable to determine whether the observed quality improvement in the CRP_LA treatment was exclusively attributable to exogenous LAB or resulted from the combined effect of CRP and LAB. Future studies must incorporate a LAB-only control group and dynamically monitor LAB colonization speed, acidification rate, and the time window of spoilage bacteria suppression, as well as physicochemical indices and microbial community succession throughout the fermentation process.

(2) Weighted gene co-expression network analysis (WGCNA) typically requires a relatively large sample size to ensure the stability of module identification. The limited number of biological replicates per group in this study may have led to unstable module partitioning or overfitting. Therefore, we regard the WGCNA results as exploratory findings rather than definitive conclusions. Future research should increase the sample size per group (*n* ≥ 6) to validate the key metabolic modules identified in this study.

(3) Through metabolomics, we identified multiple hub metabolites (e.g., zeatin, eriodictyol, *cis*-zeatin) that were significantly upregulated in the CRP_LA group; however, we could not distinguish whether these metabolites were derived directly from active components in CRP, synthesized by *oat* itself under stress conditions, or produced by LAB through biotransformation. Future studies should combine stable isotope tracing or targeted metabolite supplementation experiments to clarify the origin and functional contribution of each metabolite.

## 4. Materials and Methods

### 4.1. Raw Materials, Additives, and Experimental Design

*Oat*s (*Avena sativa* L. cv. “Mengyan No. 1”) were harvested at the milk stage from the experimental station of the Institute of Grassland Research, Chinese Academy of Agricultural Sciences. The harvested forage was wilted at room temperature to achieve a target moisture content of approximately 70% and then chopped to a uniform length of 2–3 cm. The chemical composition of the raw *oat* material is presented in Table 2.

The commercial inoculant XinLaiWang I-Straw Silage Inoculant, containing *Lactobacillus plantarum* (≥1 × 10^11^ CFU/g), *Lactobacillus buchneri* (≥1 × 10^9^ CFU/g), and *Enterococcus faecium* (≥1 × 10^9^ CFU/g), with a total viable count ≥ 1 × 10^11^ CFU/g, was obtained from XinLaiWang (Nanjing) Biotechnology Co., Ltd., Nanjing, China. Compound radix pulsatillae (CRP), a powdered mixture consisting of *Pulsatilla chinensis* (33.3%), *Fraxinus rhynchophylla* (26.7%), *Phellodendron chinense* (26.7%), and *Coptis chinensis* (13.3%), was procured from Tongrentang Pharmacy (Hohhot, China). CRP was dried at 65 °C for 6 h, ground in a grinder, and sieved through a 50-mesh screen to obtain the herbal additive. The resulting powder was sealed in zip-lock bags and stored in a cool, ventilated environment. The nutritional profile of CRP is detailed in Table 2. Three treatments were established: (1) Control (CK); (2) CRP at 40 g/kg fresh matter (4% FM); and (3) CRP plus compound LAB inoculant (CRP_LA) at 2 g/t. This application rate resulted in final silage counts of > 1 × 10^5^ CFU/g total viable bacteria, comprising *L. plantarum* (≥1 × 10^5^ CFU/g), *L. buchneri* (≥1 × 10^3^ CFU/g), and *E. faecium* (≥1 × 10^3^ CFU/g). The inclusion level of 4% CRP (fresh weight basis) was determined based on our previous pilot studies, which indicated that 4% compound *Pulsatilla chinensis* formula provided optimal ensiling performance for *oat*, simultaneously improving nutritional quality and fermentation characteristics [16].

### 4.2. Silage Preparation and Sampling

For each treatment, approximately 200 g of the chopped *oat* forage was uniformly mixed with the respective additives and tightly packed into vacuum-sealable plastic pouches. Each treatment was prepared in triplicate. The pouches were vacuum-sealed and stored at room temperature (approx. 25 °C) for 60 days to allow for anaerobic fermentation.

After the ensiling period, the bags were opened. The contents of each replicate were thoroughly mixed, and sub-samples were immediately collected for subsequent analyses: one portion was snap-frozen in liquid nitrogen and stored at −80 °C for microbial DNA extraction; a second portion was stored at −80 °C for metabolomic analysis; and the remainder was stored at −20 °C for chemical and fermentation analyses.

### 4.3. Analysis of Fermentation Quality and Nutritional Composition

The pH and organic acid concentrations were determined from aqueous silage extracts. Briefly, 20 g of silage was homogenized with 180 mL of sterile deionized water and incubated at 4 °C for 24 h. The supernatant was obtained by filtration through four layers of cheesecloth followed by filter paper. The pH was measured directly using a precision pH meter (LAQUAtwin-pH-22, Horiba, Ltd., Kyoto, Japan). Concentrations of lactic acid (LA), acetic acid (AA), propionic acid (PA), and butyric acid (BA) were quantified using high-performance liquid chromatography (HPLC, 1260 Infinity II, Agilent Technologies, Inc., Santa Clara, CA, USA). Ammonia nitrogen (AN) content was analyzed using the phenol-hypochlorite method and expressed as a percentage of total nitrogen (TN).

For nutritional composition analysis, samples were oven-dried at 65 °C for 48 h to determine dry matter (DM) content. The dried samples were then ground to pass through a 1-mm sieve. Neutral detergent fiber (NDF) and acid detergent fiber (ADF) contents were determined using an ANKOM 200i fiber analyzer (ANKOM Technology, Macedon, NY, USA). Crude protein (CP) content was calculated as TN × 6.25, with TN measured by the Kjeldahl method using an automated analyzer (F800 Automatic Kjeldahl Analyzer (Haineng Scientific Instruments Co., Ltd., Shandong, China)). Water-soluble carbohydrates (WSC) were quantified by the anthrone–sulfuric acid colorimetric method.

### 4.4. Microbial Community Analysis

Microbial genomic DNA was extracted from the frozen silage samples using the E.Z.N.A.^®^ Soil DNA Kit (Omega Bio-tek, Inc., Norcross, GA, USA), following the manufacturer’s instructions, with each extraction performed in triplicate. The integrity and concentration of the extracted DNA were checked. The hypervariable V3-V4 region of the bacterial 16S rRNA gene was amplified using the primers 799F (5′-AACMGGATTAGATACCCKG-3′) and 1193R (5′-ACGTCATCCCCACCTTCC-3′). Amplicon sequencing was performed on an Illumina MiSeq platform (Illumina, Inc., San Diego, CA, USA); sequencing service provided by Shanghai Meiji Pharmaceutical Technology Co., Ltd. (Shanghai, China).

### 4.5. Metabolomic Profiling

Untargeted metabolomic analysis was conducted by Metware Biotechnology Co., Ltd. (Jiaxing, China). Frozen silage samples were freeze-dried, and metabolites were extracted using a methanol–water solvent system. The extracts were analyzed using an ultra-performance liquid chromatography (UPLC) system coupled with tandem mass spectrometry (MS/MS). Quality control (QC) samples, prepared by mixing equal volumes of all samples, were injected at regular intervals throughout the analytical run to monitor instrument stability.

### 4.6. Statistical Analyses

#### 4.6.1. Statistical Analysis of Fermentation Quality and Nutritional Composition

Data were organized and summarized using Microsoft Excel 2019, and one-way ANOVA was performed using IBM SPSS Statistics 26. Duncan’s multiple range test was employed for multiple comparisons among treatments. Results are expressed as mean ± standard error of the mean (SEM). Significance levels were set at *p* < 0.05 for significant difference, *p* < 0.01 for highly significant difference, and *p* < 0.001 for extremely significant difference.

Based on the OTU abundance table obtained from 16S rRNA gene sequencing, diversity analysis was conducted using R software (version 4.1.0) with relevant packages (vegan, ggplot2). α-diversity indices included Coverage, Chao1, Shannon, and Simpson indices to assess microbial community richness and evenness. For β-diversity, principal coordinate analysis (PCoA) based on unweighted UniFrac and weighted UniFrac distances was used to visualize differences in microbial community structure among samples. The Kruskal–Wallis H test was used to compare the relative abundance of microbial genera across different treatment groups, screening for significantly differential genera (*p* < 0.05). Stacked bar charts were generated to display relative abundance at the phylum and genus levels, and Upset plots were constructed to illustrate unique and shared OTUs among groups.

#### 4.6.2. Metabolite Data Processing Using R Software

(1) Principal Component Analysis (PCA): Unsupervised dimensionality reduction analysis was performed on metabolite abundance across all samples to observe the overall separation trend of metabolite profiles among groups.

(2) Differential Metabolite Screening: Orthogonal partial least squares discriminant analysis (OPLS-DA) was employed. Variable importance in projection (VIP) > 1, combined with fold change (FC) criteria of FC > 2 or FC < 0.5, and Student’s *t*-test *p* < 0.05 were used as standards to screen differentially accumulated metabolites between comparison groups.

(3) KEGG Pathway Enrichment Analysis: Differential metabolites were annotated to the Kyoto Encyclopedia of Genes and Genomes (KEGG) database. Hypergeometric test was used to identify significantly enriched metabolic pathways (*p* < 0.05). Enrichment results were visualized as bubble plots, where point size represents the number of metabolites enriched in each pathway and color indicates enrichment significance.

(4) Weighted Gene Co-expression Network Analysis (WGCNA): Based on the metabolite abundance matrix, weighted co-expression network analysis was performed using the R package 4.2.2 WGCNA 1.71. Hierarchical clustering was first used to partition metabolites into distinct modules, and module clustering dendrograms and network heatmap plots were generated. The correlation between module eigengenes and treatment groups (CK, CRP, CRP_LA) as well as silage quality parameters (pH, DM, WSC, CP, NDF, ADF, LA, AA, PA, AN/TN) was calculated to identify modules significantly associated with specific treatments or quality parameters (correlation coefficient |r| > 0.6, *p* < 0.05). KEGG enrichment analysis was performed on metabolites within key modules, and hub metabolites were screened by intersecting with differential metabolites.

#### 4.6.3. Correlation Analysis

(1) Mantel Test: Mantel tests were used to analyze correlations between microbial communities (β-diversity distance matrix) and metabolite profiles (metabolite abundance distance matrix), as well as associations between key metabolites and fermentation quality indices. Results were presented as Mantel heatmaps.

(2) Network Correlation Analysis: Based on Spearman’s rank correlation coefficient, correlation networks were constructed among key metabolites, between key metabolites and fermentation quality parameters, between key metabolites and differential microbial genera, and between differential microbial genera and fermentation quality parameters. Significant correlation thresholds were set at |r| > 0.6 and *p* < 0.05. Network visualization was performed using R packages.

## 5. Conclusions

The combination of Compound Radix Pulsatillae and lactic acid bacteria (CRP_LA) improves *oat* silage quality through three pathways: (1) acidification by exogenous LAB, which establishes a selective acidic barrier; (2) enrichment of functional microbial groups driven by CRP bioactive components together with LAB; and (3) accumulation of antioxidant and antimicrobial metabolites that suppress spoilage microorganisms. This study provides a multi-omics theoretical basis for the rational design of plant-based silage additives combining herbal ingredients with LAB.

## Figures and Tables

**Table 1:** Effects of Compound Radix Pulsatillae and lactic acid bacteria on nutritional and fermentation quality of *oat* silage.

Index	CK	CRP	CRP_LA	SEM	*p*
pH	5.73 ^a^	5.01 ^b^	4.82 ^b^	0.14	0.002
DM (%)	31.29 ^c^	34.96 ^b^	36.74 ^a^	0.82	<0.001
WSC (g/kg DM)	21.21 ^c^	30.62 ^b^	37.70 ^a^	0.24	<0.001
CP (g/kg DM)	117.32 ^a^	110.43 ^b^	111.34 ^b^	0.11	<0.001
NDF (g/kg DM)	598.64 ^a^	523.41 ^b^	512.32 ^b^	1.39	0.001
ADF (g/kg DM)	357.03 ^a^	318.01 ^b^	309.84 ^b^	0.79	0.003
LA (g/kg DM)	24.22 ^c^	36.50 ^b^	47.83 ^a^	0.34	<0.001
AA (g/kg DM)	17.12 ^a^	19.50 ^a^	12.61 ^b^	0.11	0.007
PA (g/kg DM)	4.71 ^a^	3.20 ^b^	3.62 ^b^	0.02	0.001
BA (g/kg DM)	-	-	-	-	-
AN/TN (%)	7.01 ^a^	4.59 ^b^	3.79 ^c^	0.49	<0.001

DM: Dry matter; CP: Crude protein; NDF: Neutral detergent fiber; ADF: Acid detergent fiber; LA: Lactic acid; AA: Acetic acid; PA: Propionic acid; BA: Butyric Acid; AN/TN: The ratio of ammonia nitrogen to total nitrogen. Means within a row with different superscripts differ significantly (*p* < 0.05).

**Table 3:** Classification and pathway enrichment of key metabolites.

Index	Compounds	Class I	Involved Pathways	Function	Effect of Additive
pme2063	Cis-Zeatin	Alkaloids	ko00908, ko01100, ko01110	Plant cytokinin involved in regulating plant defense responses [17]; contributes to WSC and DM retention.	Not significant in CRP; strongly induced by LAB.
mws0064	Eriodictyol (5,7,3′,4′-Tetrahydroxyflavanone) *	Flavonoids	ko00941, ko01100, ko01110	Antioxidant, antibacterial, and anti-inflammatory activities [18,19].	Induced by CRP; further enhanced by LAB.
mws0884	Cyclic 3′,5′-Adenylic acid	Nucleotides and derivatives	ko00230, ko01100	Bacterial second messenger regulating gene expression; c-di-AMP plays key signaling roles in LAB; promotes LAB growth and metabolic activity [20].	Weak effect by CRP; strongly increased by LAB.
pme1474	5′-Deoxy-5′-(methylthio)adenosine	Nucleotides and derivatives	ko00270, ko00908, ko01100	Byproduct of bacterial polyamine synthesis, recycled via methionine salvage pathway; modulates LAB methylation and adaptation [21].	No effect by CRP; abundantly produced by LAB.
pma0149	Sinapoyl malate	Phenolic acids	ko00940, ko01110	Synergistic antimicrobial activity [22].	Strongly suppressed by CRP; completely reversed and super-induced by LAB.
Zaxn005652	Pisiferic acid	Terpenoids	ko00904, ko01110	Inhibitory activity against both Gram-negative and Gram-positive bacteria	Induced by CRP; further enhanced by LAB.

**Table 2:** Nutritional composition of raw materials (%) dry matter basis.

Raw Materials	DM (% FM)	WSC (g/kg DM)	CP (g/kg DM)	NDF (g/kg DM)	ADF (g/kg DM)
*Oat*	30.91	79.5	90.5	603.02	335.62
CRP	98.12	87.83	70.52	435.11	295.31

## Data Availability

The original contributions presented in this study are included in the article/Supplementary Materials. Further inquiries can be directed to the corresponding authors.

## Supplementary Materials

The following supporting information can be downloaded at https://www.mdpi.com/article/10.3390/ijms27125577/s1.

## Abbreviations

The following abbreviations are used in this manuscript:

AA	Acetic acid
ADF	Acid detergent fiber
AN	Ammonia nitrogen
AN/TN	Ammonia nitrogen to total nitrogen ratio
BA	Butyric acid
CHM	Chinese herbal medicines
CK	Control (no additive)
CP	Crude protein
CRP	Compound Radix Pulsatillae
CRP_LA	Compound Radix Pulsatillae + lactic acid bacteria inoculant
DM	Dry matter
FC	Fold change
FO	Fresh *oat* (before ensiling)
KEGG	Kyoto Encyclopedia of Genes and Genomes
LA	Lactic acid
LAB	Lactic acid bacteria
NDF	Neutral detergent fiber
OTU	Operational taxonomic unit
PA	Propionic acid
PCA	Principal component analysis
PCoA	Principal coordinate analysis
SEM	Standard error of the mean
TN	Total nitrogen
UPLC-MS/MS	Ultra-performance liquid chromatography–tandem mass spectrometry
VIP	Variable importance in projection
WGCNA	Weighted gene co-expression network analysis
WSC	Water-soluble carbohydrates
