# Supplementary material for: Multi Omics Analysis Reveals That Compound Radix Pulsatillae and Lactic Acid Bacteria Reprogram the Microbiome Metabolome Network in Oat Silage
Source: Int J Mol Sci. 2026 Jun 20;27(12):5577. doi: 10.3390/ijms27125577 (PMC13299535; doi:10.3390/ijms27125577)
Supplement: Supplementary file 1 [file ijms-27-05577-s001.zip › ijms-4357722-supplementary.pdf]

Supplementary Table S1 Metabolite classification of the main modules

Module Colors: blue

Index	Compounds	Class I	Class II
Cmyp007180	Dihydroactinidiolide	Others	Lactones
Cmyp009787	Damascenone	Others	Ketone compounds
Hahp000801	alanine betaine	Alkaloids	Alkaloids
HJAP165	N-Isobutyl-2,4-tetradecanedienamide	Alkaloids	Alkaloids
HJKP000649	N-benzylformamide*	Alkaloids	Alkaloids
Hmcp001824	Ailanindole	Alkaloids	Plumerane
Hmcp002354	Crenatine	Alkaloids	Plumerane
Hmcp003783	1-Acetyl-β-carboline	Alkaloids	Plumerane
Hmgn001653	Protocatechualdehyde*	Phenolic acids	Phenolic acids
Hmln007772	Nordihydrocapsiate	Phenolic acids	Phenolic acids
Hmmp001310	3-Indoleacrylic acid*	Alkaloids	Plumerane
Hmqn004825	Rabdosia acid A*	Lipids	Free fatty acids
Hmtp000776	4,5,6-Trihydroxy-2-cyclohexen-1-ylideneacetonitrile	Alkaloids	Alkaloids
Ladp002935	3-quinolinecarboxylic acid*	Alkaloids	Quinoline alkaloids
Lafp002342	2,3-Dihydroxy-1-(4-hydroxy-3,5-dimethoxyphenyl)propan-1-one	Phenolic acids	Phenolic acids
Lafp003256	3,4'-Dihydroxy-3',5'-dimethoxypropiophenone	Phenolic acids	Phenolic acids
Lafp005959	7-Hydroxy-costol	Terpenoids	Sesquiterpenoids
Lahp002608	3,5-Dihydro-2H-Furo[3,2-C]Quinolin-4-One*	Alkaloids	Quinoline alkaloids

Lahp003997	Glucosyl-rhamnazin-3-O-β-D-glucoside	Flavonoids	Flavonols
Lasp002993	Isobavachalcone glucoside	Flavonoids	Chalcones
Lasp011732	Ent-16beta-Methoxy-19-Kauranoic Acid	Terpenoids	Ditepenoids
Lchp000221	(Z)-3-Ethylidene-7-hydroxy-6-methoxyphthalide	Others	Others
Lhcp090204	β-Ionone	Others	Ketone compounds
Lhcp120602	Eremophila-9-en-8β,11-diol	Terpenoids	Sesquiterpenoids
Lhhp120808	Illicinone F	Lignans and Coumarins	Coumarins
Lhlp120627	Carvylacetate	Terpenoids	Monoterpenoids
Lhmp122225	dalodorin B	Terpenoids	Sesquiterpenoids
Lmbn000193	Tartronate semialdehyde*	Organic acids	Organic acids
Lmbn000198	3-Dehydro-L-Threonic Acid	Others	Saccharides
Lmbn002644	4-Methylbenzaldehyde*	Others	Aldehyde compounds
Lmbn002648	α-Hydroxycinnamic Acid*	Phenolic acids	Phenolic acids
Lmbn002737	3-Methylbenzaldehyde*	Others	Aldehyde compounds
Lmbn004685	5S,8R-DiHODE; (5S,8R,9Z,12Z)-5,8-Dihydroxyoctadeca-9,12-dienoate	Lipids	Free fatty acids
Lmbn005369	13(S)-HODE;13(S)-Hydroxyoctadeca-9Z,11E-dienoic acid*	Lipids	Free fatty acids
Lmbn005443	13-KODE; (9Z,11E)-13-Oxooctadeca-9,11-dienoic acid*	Lipids	Free fatty acids
Lmbn005662	9(10)-EpOME;(9R,10S)-(12Z)-9,10-Epoxyoctadecenoic acid	Lipids	Free fatty acids
Lmbn005923	Crepenynic acid*	Lipids	Free fatty acids
Lmbn006152	(9Z,11E)-Octadecadienoic acid*	Lipids	Free fatty acids
Lmbn007891	Hydroxy ricinoleic acid	Lipids	Free fatty acids

Lmcn009539	E,E,Z-1,3,12-Nonadecatriene-5,14-diol	Lipids	Free fatty acids
Lmcp003522	Baldrinal	Terpenoids	Monoterpenoids
Lmgn002679	Phenol	Phenolic acids	Phenolic acids
Lmgp000659	2-Phenylacetamide*	Alkaloids	Alkaloids
Lmgp004217	Diosmetin-8-C-(2''-O-rhamnosyl)glucoside	Flavonoids	Flavones
Lmgp004784	Apiferol	Flavonoids	Flavones
Lmhp112042	1-Linoleoylglycerol	Lipids	Glycerol ester
Lmjn005592	Cirsilineol (4',5-Dihydroxy-3',6,7-trimethoxyflavone)	Flavonoids	Flavanones
Lmlp002205	Isololiolide	Terpenoids	Terpene
Lmlp003161	N-Feruloylputrescine	Alkaloids	Phenolamine
Lmmn001643	2-Hydroxycinnamic acid*	Phenolic acids	Phenolic acids
Lmmn002164	Monomethyl succinate*	Organic acids	Organic acids
Lmmp006540	Tricin-7-O-(2''-Sinapoyl)glucuronide	Flavonoids	Flavones
Lmnp002741	Genkwanin-6-C-(2''-O-apiosyl)glucoside	Flavonoids	Flavones
Lmqp010784	Progesterone	Terpenoids	Ditepenoids
Lmrj001698	L-Seryl-L-Isoleucine	Amino acids and derivatives	Amino acids and derivatives
Lmsn004901	Tetracosanoic Acid (Lignoceric acid)	Lipids	Free fatty acids
Lmsp004851	3-Hydroxyphloretin	Flavonoids	Chalcones
Lmtp002474	Apigenin-6-C-(2''-glucosyl)arabinoside	Flavonoids	Flavones
Lmyn007883	9,16-Dihydroxypalmitic acid	Lipids	Free fatty acids
Lmyp002912	2-Hydroxy-7-methoxy-1,4-benzoxazin-3(2H)-one (HMBOA)	Alkaloids	Alkaloids
Lmyp003951	3-Hydroxy-1-(4-hydroxy-3,5-dimethoxyphenyl)propan-1-one	Phenolic acids	Phenolic acids

Lmzn001925	3,4'-Dihydroxy-3'-methoxybenzenepentanoic acid	Phenolic acids	Phenolic acids
Lssp210086	Balanophonin B	Lignans and Coumarins	Lignans
MA10074217	4-Hydroxyquinoline	Alkaloids	Quinoline alkaloids
MA10107783	3-[(1-Carboxyvinyl)oxy]benzoic acid	Phenolic acids	Phenolic acids
MEDL01869	Methylgingerol	Phenolic acids	Phenolic acids
ML10172161	Hydroxypyruvic acid*	Organic acids	Organic acids
MW0010749	(+)-7-iso-Jasmonic acid*	Organic acids	Organic acids
MW0011652	4-Isopropenylcyclohexene-1-carboxylic acid	Organic acids	Organic acids
MW0012222	12S-HHT	Lipids	Free fatty acids
MW0012501	15S-hydroperoxy-11Z,13E-eicosadienoic acid	Lipids	Free fatty acids
MW0012595	7,8,17-trihydroxy-4,9,11,13,15,19-docosahexaenoic acid	Lipids	Free fatty acids
MW0014613	5(S)-HETrE	Organic acids	Organic acids
MW0015406	(-)-9,10-Dihydrojasmonic acid	Organic acids	Organic acids
MW0017099	9s,13r-12-Oxophytodienoic Acid	Lipids	Free fatty acids
MW0052697	Ethyl (±)-3-hydroxyoctanoate	Others	Lactones
MW0052740	Ethyl tetradecanoate	Others	Lactones
MW0054671	Malyngamide H	Others	Ketone compounds
MW0062117	trans-p-Menthane-1,8-diol	Terpenoids	Terpene
MW0103070	Valerenic acid	Terpenoids	Sesquiterpenoids
MW0103813	(±)-3-Hydroxynonanoic acid	Organic acids	Organic acids
MW0103898	Phenylpropionylglycine	Organic acids	Organic acids
MW0103903	3-Hydroxydodecanoic acid	Organic acids	Organic acids

MW0104373	2-[1-(Carboxymethyl)-3-methylcyclohexyl]acetic acid	Organic acids	Organic acids
MW0105006	1,3,4-Trihydroxy-5-oxocyclohexane-1-carboxylic acid	Organic acids	Organic acids
MW0105037	3-Hydroxydecanoic acid	Organic acids	Organic acids
MW0106498	3-Hydroxyoctanoic acid	Organic acids	Organic acids
MW0107570	Ile-Met	Amino acids and derivatives	Amino acids and derivatives
MW0107988	Lysyl-alanyl-alanine	Amino acids and derivatives	Amino acids and derivatives
MW0108646	N-Acetylproline	Amino acids and derivatives	Amino acids and derivatives
MW0109142	Phe-Val	Amino acids and derivatives	Amino acids and derivatives
MW0109201	Phenylalanylcysteine	Amino acids and derivatives	Amino acids and derivatives
MW0114605	7-Hydroxy-3,7-dimethyloctanal	Others	Aldehyde compounds
MW0117723	2-(N-Morpholino)ethanesulfonic acid	Organic acids	Organic acids
MW0119612	3-Hydroxy-3-methyloxindole	Alkaloids	Plumerane
MW0120419	4-Cyanoindole	Alkaloids	Plumerane
MW0131241	3-(3-hydroxy-4-methoxyphenyl)propanoic acid	Organic acids	Organic acids
MW0131473	5,7,3',4'-Tetrahydroxy-6,8-dimethoxyflavone	Flavonoids	Flavones
MW0144650	Ala-Leu-Val-Arg	Amino acids and derivatives	Amino acids and derivatives
MW0146139	Asp-Ile-Asn-Lys	Amino acids and derivatives	Amino acids and derivatives

MW0148031	Cys-Leu-Trp	Amino acids and derivatives	Amino acids and derivatives
MW0150834	His-Arg-Gly	Amino acids and derivatives	Amino acids and derivatives
MW0150898	His-Gln-Glu-Asp	Amino acids and derivatives	Amino acids and derivatives
MW0151013	His-Lys-Ile-Asp	Amino acids and derivatives	Amino acids and derivatives
MW0152471	Leu-Pro-Phe	Amino acids and derivatives	Amino acids and derivatives
MW0152574	Leu-Val-Val-Val-Gly	Amino acids and derivatives	Amino acids and derivatives
MW0153757	Met-Glu	Amino acids and derivatives	Amino acids and derivatives
MW0154297	Nap-Tyr-OH	Amino acids and derivatives	Amino acids and derivatives
MW0155161	Phe-Gln-His	Amino acids and derivatives	Amino acids and derivatives
MW0155194	Phe-Gly-Glu	Amino acids and derivatives	Amino acids and derivatives
MW0156666	Ser-Asn-Ser	Amino acids and derivatives	Amino acids and derivatives
MW0168514	2-Hydroxymyristic acid	Organic acids	Organic acids
mws0008	Hydrocinnamic acid*	Phenolic acids	Phenolic acids
mws0089	Kaempferol-7-O-glucoside	Flavonoids	Flavonols
mws0102	Indole-5-carboxylic acid*	Alkaloids	Plumerane
mws0103	Indole-3-carboxaldehyde	Alkaloids	Plumerane
mws0147	β-Hydroxyisovaleric acid	Organic acids	Organic acids

mws0154	Shikimic acid	Organic acids	Organic acids
mws0232	Riboflavin (Vitamin B2)	Others	Vitamin
mws0250	L-Tyrosine	Amino acids and derivatives	Amino acids and derivatives
mws0260	L-Arginine	Amino acids and derivatives	Amino acids and derivatives
mws0267	DL-Glyceric Acid	Organic acids	Organic acids
mws0277	Quinic Acid	Organic acids	Organic acids
mws0282	L-Tryptophan	Amino acids and derivatives	Amino acids and derivatives
mws0336	Urocanic acid	Organic acids	Organic acids
mws0367	α-Linolenic Acid*	Lipids	Free fatty acids
mws0396	Elaidic Acid*	Lipids	Free fatty acids
mws0397	Cis-5,8,11,14,17-Eicosapentaenoic Acid	Lipids	Free fatty acids
MWS04412	N(6),N(6)-Dimethyl-L-lysine	Amino acids and derivatives	Amino acids and derivatives
MWS04559g	3-Hydroxy-L-phenylalanine	Amino acids and derivatives	Amino acids and derivatives
mws0458	Vanillin; 4-Hydroxy-3-Methoxybenzaldehyde*	Phenolic acids	Phenolic acids
mws0489	Benzoylformic acid	Organic acids	Organic acids
mws0520	N-Acetyl-L-tyrosine	Amino acids and derivatives	Amino acids and derivatives
MWS0559	1,6-anhydro-β-D-glucose	Others	Saccharides
mws0574	2-Hydroxyisobutyric acid	Organic acids	Organic acids
mws0576	3-Hydroxybutyric acid	Organic acids	Organic acids

MWS0813	5-Oxoproline	Amino acids and derivatives	Amino acids and derivatives
mws0885	2,4-Dihydroxybenzoic acid	Phenolic acids	Phenolic acids
mws0913	Kaempferol-3-O-galactoside (Trifolin)	Flavonoids	Flavonols
mws0972	6-Hydroxyhexanoic acid	Organic acids	Organic acids
mws0984	L-Sepiapterin	Nucleotides and derivatives	Nucleotides and derivatives
mws1105	Gramine	Alkaloids	Plumerane
mws1292	Isoschaftoside	Flavonoids	Flavones
mws1417	Indole-3-carboxylic acid*	Alkaloids	Plumerane
mws1488	Palmitic acid	Lipids	Free fatty acids
mws1491	Linoleic acid*	Lipids	Free fatty acids
mws1550	S-Allyl-L-cysteine	Amino acids and derivatives	Amino acids and derivatives
mws1587	L-Norleucine*	Amino acids and derivatives	Amino acids and derivatives
mws1715	Cordycepin (3'-Deoxyadenosine)*	Nucleotides and derivatives	Nucleotides and derivatives
MWS1840	Triethylamine	Alkaloids	Alkaloids
MWS1848	Phenyl acetate	Phenolic acids	Phenolic acids
MWS1983	Maltitol	Others	Saccharides
MWS201381	Ser-Trp	Amino acids and derivatives	Amino acids and derivatives
MWS201391	Asn-Ile	Amino acids and derivatives	Amino acids and derivatives
MWS201449	Asn-Leu	Amino acids and derivatives	Amino acids and derivatives

MWS20194	Cinnamic acid	Phenolic acids	Phenolic acids
MWS2032	6-Methylnicotinamide	Alkaloids	Pyridine alkaloids
mws2124	2-Phosphoglycolate	Organic acids	Organic acids
mws4113	3'-Hydroxy-4'-O-methylglabridin	Flavonoids	Other Flavonoids
mws4176	L-Alanyl-L-Phenylalanine	Amino acids and derivatives	Amino acids and derivatives
MWS4305	Erythrose	Others	Saccharides
MWS4471	N-Alpha-Acetyl-L-Asparagine	Amino acids and derivatives	Amino acids and derivatives
MWS4519	4-Methylhippuric Acid	Amino acids and derivatives	Amino acids and derivatives
MWS4533	2-Phenylpropionic Acid*	Organic acids	Organic acids
mws5041	L-Glycyl-L-isoleucine	Amino acids and derivatives	Amino acids and derivatives
MWS5231	Tridecanedioic acid	Lipids	Free fatty acids
MWSCX015	Caffeic aldehyde	Phenolic acids	Phenolic acids
MWSCX017	4-hydroxyphenyl acrylaldehyde	Others	Aldehyde compounds
MWSHC20102	3-Hydroxy-1-(4-Hydroxy-3-Methoxyphenyl)Propan-1-One	Phenolic acids	Phenolic acids
MWSHC20122	Dehydrovomifoliol	Terpenoids	Sesquiterpenoids
MWSHC20196	10α-Hydroxy-cadin-4-en-15-al	Terpenoids	Sesquiterpenoids
MWSHC2083	1β,6α-Dihydroxy-4(14)-eudesmene	Others	Others
MWSHC2099	Salcolin A*	Flavonoids	Flavones
MWSmce001	5-Acetylsalicylic acid	Phenolic acids	Phenolic acids
MWSmce040	Isovanillin	Phenolic acids	Phenolic acids
MWSmce098	Nonivamide	Alkaloids	Phenolamine

MWSmce183	D-Mandelic acid	Organic acids	Organic acids
MWSmce190	4-Hydroxy-L-Isoleucine	Amino acids and derivatives	Amino acids and derivatives
MWSmce220	D-Glucono-1,5-lactone	Others	Saccharides
MWSmce257	D-Pantothenic Acid	Others	Vitamin
MWSmce284	3,4-Dimethoxyphenol	Phenolic acids	Phenolic acids
MWSmce332	3,4-Dimethoxycinnamic acid	Phenolic acids	Phenolic acids
MWSmce460	2-Piperidone	Alkaloids	Piperidine alkaloids
MWSmce632	Vanillin acetate	Phenolic acids	Phenolic acids
MWSmce692	γ-Linolenic Acid*	Lipids	Free fatty acids
MWSslk080	10-Hydroxydecanoic acid	Lipids	Free fatty acids
MWSslk083	4-Hydroxy-3,5-dimethoxybenzyl alcohol	Phenolic acids	Phenolic acids
MWSslk133	1,14-Tetradecanedioic Acid	Lipids	Free fatty acids
MWSslk183	7,8-Dihydroxy-4-phenylcoumarin	Lignans and Coumarins	Coumarins
MWSslk225	1-Indanone	Others	Ketone compounds
MWStz070	N-(2-Hydroxy-4-methoxyphenyl)acetamide	Alkaloids	Phenolamine
pmb0128	δ-Tridecalactone	Lipids	Free fatty acids
pmb0490	p-Coumaroylputrescine	Alkaloids	Phenolamine
pmb0744	Tricin-4'-O-Benzoic Acid	Flavonoids	Flavones
pmb0745	Tricin-4'-O-syringyl alcohol	Flavonoids	Flavones
pmb0746	Tricin-4'-O-(guaiacylglycerol)ether*	Flavonoids	Flavones
pmb0764	4-Methyl-5-thiazoleethanol	Others	Others
pmb0818	Methoxyindoleacetic acid	Alkaloids	Plumerane
pmb0889	Punicic acid (9Z,11E,13Z-octadecatrienoic acid)	Lipids	Free fatty acids

pmb0962	L-Lysine-Butanoic Acid	Amino acids and derivatives	Amino acids and derivatives
pmb1574	9,12-Octadecadien-6-Ynoic Acid	Lipids	Free fatty acids
pmb1587	4-Hydroxy-3,5-diisopropylbenzaldehyde	Phenolic acids	Phenolic acids
pmb2221	4-Hydroxysphinganine	Lipids	Sphingolipids
pmb2640	Dodecanoic acid (Lauric acid)	Lipids	Free fatty acids
pmb2786	9-Hydroxy-10,12,15-octadecatrienoic acid*	Lipids	Free fatty acids
pmb2787	9-Oxo-10E,12Z-octadecadienoic acid*	Lipids	Free fatty acids
pmb2789	13S-Hydroperoxy-6Z,9Z,11E-octadecatrienoic acid	Lipids	Free fatty acids
pmb2791	9-Hydroperoxy-10E,12,15Z-octadecatrienoic acid	Lipids	Free fatty acids
pmb2799	12,13-Epoxy-9-Octadecenoic Acid	Lipids	Free fatty acids
pmb2804	13S-Hydroperoxy-9Z,11E-octadecadienoic acid	Lipids	Free fatty acids
pmb3000	Chrysoeriol-7-O-(6"-acetyl)glucoside	Flavonoids	Flavones
pmb3031	Tricin-4'-O-glycerol	Flavonoids	Flavones
pmb3032	Tricin-7-O-(2"-Malonyl)rhamnoside	Flavonoids	Flavones
pmb3053	Tricin-4'-O-eudesmic acid	Flavonoids	Flavones
pme0006	L-Proline*	Amino acids and derivatives	Amino acids and derivatives
pme0008	L-Citrulline	Amino acids and derivatives	Amino acids and derivatives
pme0021	L-Phenylalanine	Amino acids and derivatives	Amino acids and derivatives

pme0026	L-Lysine	Amino acids and derivatives	Amino acids and derivatives
pme0124	L-Glycyl-L-proline	Amino acids and derivatives	Amino acids and derivatives
pme0183	Isoguanine	Nucleotides and derivatives	Nucleotides and derivatives
pme0193	L-Glutamine	Amino acids and derivatives	Amino acids and derivatives
pme0241	Benzoic acid	Phenolic acids	Phenolic acids
pme0253	N-Acetyl-L-leucine	Amino acids and derivatives	Amino acids and derivatives
pme0264	Thymidine	Nucleotides and derivatives	Nucleotides and derivatives
pme0295	4-Acetamidobutyric acid	Organic acids	Organic acids
pme0428	3,4,5-Trimethoxycinnamic acid	Phenolic acids	Phenolic acids
pme1002	L-Tyramine	Alkaloids	Alkaloids
pme1119	Inosine	Nucleotides and derivatives	Nucleotides and derivatives
pme1184	2'-Deoxyguanosine	Nucleotides and derivatives	Nucleotides and derivatives
pme1194	2'-Deoxycytidine	Nucleotides and derivatives	Nucleotides and derivatives
pme1439	p-Coumaric acid	Phenolic acids	Phenolic acids
pme1654	Jasmonic acid*	Organic acids	Organic acids
pme1975	Malonic acid	Organic acids	Organic acids
pme2244	3-Indolepropionic acid	Alkaloids	Plumerane
pme2266	Biotin	Others	Vitamin

pme2362	Mandelic acid	Organic acids	Organic acids
pme2527	L-Ornithine	Amino acids and derivatives	Amino acids and derivatives
pme2596	4-Pyridoxic acid	Others	Vitamin
pme2996	4-Hydroxycoumarin	Lignans and Coumarins	Coumarins
pme3005	N,N-Dimethylformamide	Alkaloids	Alkaloids
pme3011	γ-Aminobutyric acid	Organic acids	Organic acids
pme3083	2-(Formylamino)benzoic acid	Phenolic acids	Phenolic acids
pme3351	Allysine(6-Oxo DL-Norleucine)	Amino acids and derivatives	Amino acids and derivatives
pme3961	2'-Deoxyadenosine*	Nucleotides and derivatives	Nucleotides and derivatives
pmf0174	1-Decanol*	Others	Alcohol compounds
pmf0397	Arachidic acid	Lipids	Free fatty acids
pmf0557	Orange I	Alkaloids	Alkaloids
pmn001491	Dendrobiumane D	Others	Others
pmn001684	Tuberonic Acid	Organic acids	Organic acids
pmn001686	10,16-Dihydroxypalmitic acid	Lipids	Free fatty acids
pmn001689	9-Hydroxy-12-oxo-15(Z)-octadecenoic acid	Lipids	Free fatty acids
pmn001691	9,12,13-Trihydroxy-10,15-octadecadienoic acid	Lipids	Free fatty acids
pmp000086	1-Feruloyl-sn-glycerol	Phenolic acids	Phenolic acids
pmp000087	2-Feruloyl-sn-glycerol	Phenolic acids	Phenolic acids
pmp000127	Chrysoeriol-6,8-di-C-glucoside	Flavonoids	Flavones
pmp000235	Salcolin B*	Flavonoids	Flavones

pmp000263	3-Hydroxyurs-12,19-dien-28-oic acid (Sanguisorbigenin)	Terpenoids	Triterpene
pmp000970	Hispanolone	Terpenoids	Ditepenoids
pmp001235	Cnidilide	Others	Lactones
Qmjp080402	(11E)-(4S,5R,9S,10S)-15,16-Bisnor-13-oxo-8(17),11-labdadien-19-ol	Terpenoids	Sesquiterpenoids
Qmyp101119	Procurcumenol	Terpenoids	Sesquiterpenoids
Qmyp101137	(Z)-4-[3-(β-D-Glucopyranosyloxy)butylidene]-3,5,5-trimethyl-2-cyclohexen-1-one(ISO1)	Terpenoids	Sesquiterpenoids
Qmyp101403	11,12-O-Isopropyfidenesolajiangxin F	Terpenoids	Sesquiterpenoids
Qmyp101406	JiangxiBaiyingsu H	Terpenoids	Sesquiterpenoids
Qmyp101439	Blumenol C	Terpenoids	Sesquiterpenoids
Rfmb087	9-Hydroxy-13-oxo-10-octadecenoic Acid	Lipids	Free fatty acids
Rfmb091	9S-Hydroxy-10E,12Z-octadecadienoic acid*	Lipids	Free fatty acids
Rfmb320	1-Methylpiperidine-2-carboxylic acid	Organic acids	Organic acids
Sacp006536	epiacorone	Terpenoids	Sesquiterpenoids
Smjp001258	Abrine	Alkaloids	Plumerane
Wadp009201	7-Oxoabietic acid	Terpenoids	Ditepenoids
Wafn011571	alpha-Hydroxylinoleic acid*	Lipids	Free fatty acids
Wbjp001169	o-Carboxy-5-hydroxytryptamine	Alkaloids	Alkaloids
Wbjp001317	vasicinone	Alkaloids	Alkaloids
Wbkp003862	Flazin	Alkaloids	Alkaloids
Wbkp004013	shinjudilactone	Terpenoids	Ditepenoids
Wbmn011269	Vitexilactone	Terpenoids	Ditepenoids

Wbsp001938	Casuarine Analogue	Alkaloids	Alkaloids
Wbsp008682	serrat-14-ene-3,20,24,29-tetrol	Terpenoids	Triterpene
Wbtn014060	Nigrolineaxanthone K	Flavonoids	Other Flavonoids
Wcgn002300	Isobiflorin	Others	Ketone compounds
Wcgp010223	3-(Cyclohexen-1-yl)-2-hydroxy-4a,5-dimethyl-2,3,4,5,6,7,8,8a-octahydronaphthalen-1-one	Others	Others
Wcgp010436	3-(Cyclohexen-1-yl)-2-hydroxy-4a,5-dimethyl-2,3,4,5,6,8a-hexahydronaphthalen-1-one	Others	Others
Wcgp010745	3-(4-hydroxycyclohex-1-en-1-yl)-4a,5-dimethyl-3,4,4a,5,6,8a-hexahydronaphthalen-1(2H)-one	Others	Others
Wcsn010254	4-[3-(4,8-dimethylnona-3,7-dienyl)-3-methyloxiran-2-yl]butan-2-one	Others	Others
Wcsn011332	Ethyl (10Z,13Z)-hexadeca-10,13-dienoate	Others	Others
Wcsp009077	(6E,10E)-12-hydroxy-2,6,10-trimethyltrideca-2,6,10-trien-4-one	Others	Others
Wcsp009602	(5E,9E)-6,10,14-trimethylpentadeca-5,9,13-triene-2,12-dione	Others	Others
Xmyn008071	Gnetifolin B*	Flavonoids	Flavones
Yacp000453	3-hydroxy-1-methylpyrrolidin-2-one	Alkaloids	Pyrrole alkaloids
Yalp005502	Melazolide B	Terpenoids	Monoterpenoids
Yalp008047	Methyl 12-phenyldodecanoate	Lipids	Free fatty acids
Yamp002343	1-[2-(Furan-2-yl)-2-oxoethyl]piperidin-2-one	Alkaloids	Alkaloids
Zadn009075	Hydroperoxylinoleic acid	Lipids	Free fatty acids

Zahn004036	2,3-Dihydro-1,4-naphthoquinone	Quinones	Quinones
Zahp006071	N-(sec-butyl)tetradeca-2,4,8-trienamide	Alkaloids	Alkaloids
Zahp011689	2,2-dimethylchromene-6-carboxylic acid*	Others	Others
Zamp007073	4'-Methoxyacetophenone	Phenolic acids	Phenolic acids
Zbln013951	1-(3'-methoxy-4'-hydroxybenzyl)-2,7-dihydroxy-4-methoxyphenanthrene	Quinones	PhenAnthraquinones
Zblp001009	3-pyridine-methanol-O-β-D-glucopyranosyl	Alkaloids	Pyridine alkaloids
Zbzp007301	2-Methoxy-6-undecyl-1,4-benzoquinone	Quinones	Quinones
Zbzp007397	5,6,7,7a-tetrahydro-4,4,7a-trimethyl-2(4H)-benzofuranone	Others	Others
zjbp111502	Ciwujiatone	Lignans and Coumarins	Lignans
Zjcp102001	Diosgenin-3-O-rhamnosyl(1→3)glucoside (Polyphyllin C)	Steroids	Steroidal saponins
Zjcp102106	nuatigenin-Rha-Glc-Glc	Steroids	Steroidal saponins
Zjxp110301	3-Hydroxy-3,7,11-trimethyldodeca-1,6E,10-trien-9-yl isobutyrate	Terpenoids	Sesquiterpenoids
Zjyp102908	Ligucyperonol	Terpenoids	Sesquiterpenoids
Zjyp110109	oxyphyllone F	Terpenoids	Sesquiterpenoids
Zmbp002538	1-Methoxy-indole-3-acetamide	Alkaloids	Plumerane
Zmdp001663	γ-glutamylmethionine	Amino acids and derivatives	Amino acids and derivatives
Zmgn002106	N-Acetyl-L-phenylalanine	Amino acids and derivatives	Amino acids and derivatives
Zmgn004439	Decanoic acid	Organic acids	Organic acids
Zmgn004894	Methyl 4-hydroxybenzoate*	Phenolic acids	Phenolic acids
Zmhn002909	6-O-Glucosyl-caffeoylbenzoic acid	Phenolic acids	Phenolic acids

Zmjn004133	9S-Hydroperoxy-10E,12Z-octadecadienoic acid	Lipids	Free fatty acids
Zmmp002106	4-methyl-1,5,2,3-dioxadiazinan-2-amine	Nucleotides and derivatives	Nucleotides and derivatives
Zmmp002661	(3-hydroxybutyl)-L-leucine	Amino acids and derivatives	Amino acids and derivatives
Zmpn003368	13S-Hydroxy-9Z,11E,15Z-octadecatrienoic acid	Lipids	Free fatty acids
Zmsp002451	5-Acetyl-2,3-dihydro-6,7-dimethyl-1H-pyrrolizine	Alkaloids	Pyrrole alkaloids
Zmtn001624	N-Acetylisatin*	Alkaloids	Plumerane
Zmxp004503	Tricin-5,7-O-diglucoside	Flavonoids	Flavones
Zmyn000155	N-α-Acetyl-L-ornithine	Amino acids and derivatives	Amino acids and derivatives
Zmyn004548	12-Oxo-phytodienoic acid	Lipids	Free fatty acids
Zmyn004676	17-Hydroxylinolenic acid	Lipids	Free fatty acids
Zmyn004732	2R-hydroxy-9Z,12Z,15Z-octadecatrienoic acid	Lipids	Free fatty acids
Zmyn014236	2-Dodecoxyethyl Hydrogen Sulfate	Phenolic acids	Phenolic acids

Module Colors: brown

Index	Compounds	Class I	Class II
Cmmn012461	Dehydroabietic acid	Terpenoids	Ditepenoids
HJAP012	Luteolin-8-C-arabinoside	Flavonoids	Flavones
HJAP075	5,8-Epidioxyergosta-6,22-dien-3-ol (Ergosterol peroxide)	Steroids	Steroid
Hmdn006855	3,4-Cycloopen-cycloaltin-4(29),25-diene-24(R)-hydroxy-3-carboxylic acid	Terpenoids	Triterpene
Hmhp011220	10,11-Dihydroxy-N-(2-hydroxy-2-methylpropyl)-2,6,8-dodecatrienam-Ide	Alkaloids	Alkaloids
Hmqp005411	9-Oxo-12Z-Octadecenoic acid	Lipids	Free fatty acids
Hmqp005412	12-Hydroxyabietic Acid	Terpenoids	Ditepenoids
Hmqp006221	13-Hydroxy-9Z,11E-octadecadienoic acid	Lipids	Free fatty acids
Hmsp004517	[4]-Gingerol	Phenolic acids	Phenolic acids
Hmsp005892	[4]-Shogaol	Phenolic acids	Phenolic acids
Hmyn001360	Doitungbiphenyl A	Phenolic acids	Phenolic acids
Hmyp002656	Methyl dioxindole-3-acetate	Alkaloids	Plumerane
HX1402	peucedanol methylether	Lignans and Coumarins	Coumarins
Jmzn003632	Manglieside D	Lignans and Coumarins	Lignans
Lacp001298	4-Hydroxy-3-methoxy-β-phenethylamine	Alkaloids	Phenolamine
Latp003151	4-(Trans)-acetul-3,6,8-trihydroxy-3-methyl-dihydronapht halenone glucoside	Others	Chromone
Latp010375	Dihydroisoalantolactone	Terpenoids	Sesquiterpenoids
Lazn004796	Demethyl-erythro-Guaiacylglycerol β-Sinapyl Ether	Lignans and Coumarins	Lignans
Lcyp000031	Avenanthramide O	Alkaloids	Phenolamine

Lcyp000032	Avenanthramide P	Alkaloids	Phenolamine
Lcyp000035	Avenanthramide N	Alkaloids	Phenolamine
Lcyp000037	Avenanthramide H	Alkaloids	Phenolamine
Lcyp000039	Avenanthramide R	Alkaloids	Phenolamine
Lcyp000045	Desdiglucoavenacin A-1	Terpenoids	Triterpene Saponin
Lcyp000673	Prudomestin	Flavonoids	Flavonols
Lhmp122224	rel-(3S,6R,7S)-3,7,11-trimethyl-3,6-epoxy-1-dodecen-7,11-diol	Terpenoids	Sesquiterpenoids
Lmbn004240	9,10-Dihydroxy-12,13-epoxyoctadecanoic acid	Lipids	Free fatty acids
Lmbn005172	2,6-Dimethoxybenzaldehyde*	Phenolic acids	Phenolic acids
Lmbn009444	Ricinoleic acid	Lipids	Free fatty acids
Lmhp002031	L-Leucyl-L-Leucine*	Amino acids and derivatives	Amino acids and derivatives
Lmhp005550	N,N'-Diferuloylputrescine	Alkaloids	Phenolamine
Lmmp001410	Dihydrocaffeoylputrescine	Alkaloids	Phenolamine
Lmmp002013	Dihydroferuloylputrescine	Alkaloids	Phenolamine
Lmnp002845	Hispidulin-8-C-glucoside	Flavonoids	Flavones
Lmqn008288	3-Hydroxyoctadecanoic Acid	Lipids	Free fatty acids
Lmqn009304	Eucalyptin (5-Hydroxy-7,4'-dimethoxy-6,8-dimethylflavone)	Flavonoids	Flavanones
Lmqp006559	2-Amino-1,3-eicosanediol	Others	Alcohol compounds
Lmrn003000	2-Hydroxy-3-phenylpropanoic acid	Phenolic acids	Phenolic acids
Lmrn003201	Indole-3-lactic acid	Alkaloids	Plumerane
Lmyn007622	9,10,18-Trihydroxystearic acid	Lipids	Free fatty acids

Lmzp004885	Tricin (5,7,4'-Trihydroxy-3',5'-dimethoxyflavone)	Flavonoids	Flavones
MW0010795	14,15-Epoxy-5,8,11-eicosatrienoic acid	Lipids	Free fatty acids
MW0014259	3-Oxooctadecanoic acid	Organic acids	Organic acids
MW0014837	5S,6R-DiHETE	Lipids	Free fatty acids
MW0049085	Curcumol	Terpenoids	Sesquiterpenoids
MW0055437	Octadecanedioic acid	Lipids	Free fatty acids
MW0107567	Ile-Ile*	Amino acids and derivatives	Amino acids and derivatives
MW0107807	Leucylvaline	Amino acids and derivatives	Amino acids and derivatives
MW0123524	Dehydro-L-(+)-ascorbic acid dimer	Organic acids	Organic acids
MW0136864	7,8-Dihydroxy-4-methylcoumarin	Lignans and Coumarins	Coumarins
MW0137992	Dimethoxycurcumin	Phenolic acids	Phenolic acids
MW0139490	[6-[2-(3,4-Dihydroxyphenyl)-5,7-dihydroxy-4-oxochromen-3-yl]oxy-3,4,5-trihydroxyoxan-2-yl]methyl acetate	Flavonoids	Flavones
MW0142333	2-docosanamidoethanesulfonic acid	Organic acids	Organic acids
MW0143625	5-Hydroxyicosa-6,8,11-trienoic acid	Organic acids	Organic acids
MW0146329	Asp-Abu-OH	Amino acids and derivatives	Amino acids and derivatives
MW0152470	Leu-Pro-Ile	Amino acids and derivatives	Amino acids and derivatives
MW0152919	Lys-Cys-Asp	Amino acids and derivatives	Amino acids and derivatives
MW0153300	Madecassic acid	Terpenoids	Triterpene

MW0153521	Met-Asp-Ser	Amino acids and derivatives	Amino acids and derivatives
MW0155981	Pro-Pro-Phe	Amino acids and derivatives	Amino acids and derivatives
MW0156788	Ser-Ile-Ala-Asp	Amino acids and derivatives	Amino acids and derivatives
MW0168453	17-Octadecynoic acid	Lipids	Free fatty acids
MW0168536	2-Tridecenal	Others	Others
MWS00275g	DL-O-tyrosine	Amino acids and derivatives	Amino acids and derivatives
mws0061	Quercetin-3-O-galactoside (Hyperin)*	Flavonoids	Flavonols
mws0064	Eriodictyol (5,7,3',4'-Tetrahydroxyflavanone)*	Flavonoids	Flavanones
mws0072	Apigenin-5-O-glucoside	Flavonoids	Flavones
mws0098	Indole-2-carboxylic acid	Alkaloids	Plumerane
mws0126	LysoPC 18:0	Lipids	LPC
mws0133	Nicotinamide	Others	Vitamin
mws0159	Phenylpyruvic acid	Organic acids	Organic acids
mws0237	Azelaic acid	Organic acids	Organic acids
mws0251	Thymine	Nucleotides and derivatives	Nucleotides and derivatives
MWS0274	DL-3-Phenyllactic acid*	Organic acids	Organic acids
mws0275	L-Malic acid	Organic acids	Organic acids
mws0341	2-Hydroxyisocaproic acid	Organic acids	Organic acids
mws0344	D-Xylonic acid	Others	Saccharides
mws0467	3-(4-Hydroxyphenyl)-propionic acid*	Phenolic acids	Phenolic acids

mws0582	S-(Methyl)glutathione	Amino acids and derivatives	Amino acids and derivatives
mws0596	3-Hydroxyanthranilic acid	Alkaloids	Phenolamine
mws0612	3,4-Dimethoxyphenyl acetic acid	Phenolic acids	Phenolic acids
mws0620	N-Methyltryptamine	Alkaloids	Plumerane
mws0854	Rhamnose*	Others	Saccharides
mws0884	Cyclic 3',5'-Adenylic acid	Nucleotides and derivatives	Nucleotides and derivatives
mws1320	Tryptophol	Alkaloids	Plumerane
mws1346	DL-2-Aminoadipic acid	Alkaloids	Alkaloids
mws1434	Apigenin-6-C-glucoside (Isovitexin)*	Flavonoids	Flavones
mws1515	α-Ionone	Others	Ketone compounds
mws1595	D-Fucose*	Others	Saccharides
MWS1900	Undecanedioic acid	Lipids	Free fatty acids
MWS20182	Dihydroferulic Acid	Phenolic acids	Phenolic acids
MWS20184	[6]-Shogaol	Phenolic acids	Phenolic acids
MWS20185	[6]-Gingerol	Phenolic acids	Phenolic acids
MWS2099	4-Methylbenzoic acid	Phenolic acids	Phenolic acids
MWS2376	4-Hydroxycyclohexylcarboxylic acid	Organic acids	Organic acids
mws2623	11-Octadecanoic acid(Vaccenic acid)*	Lipids	Free fatty acids
mws4085	Sinapic acid	Phenolic acids	Phenolic acids
mws5045	12-Hydroxydodecanoic acid	Lipids	Free fatty acids
MWS5235	12-Hydroxyoctadecanoic acid	Lipids	Free fatty acids
MWSCX010	Humula-3(12),7(13),9(E)-triene-2,6-diol	Terpenoids	Sesquiterpenoids
MWSHY0009	Diosmetin (5,7,3'-Trihydroxy-4'-methoxyflavone)*	Flavonoids	Flavones

MWSmce016	Nicotinic Acid Methyl Ester(Methyl Nicotinate)	Alkaloids	Pyridine alkaloids
MWSmce165	L-Fucitol	Others	Saccharides
MWSmce314	Oxindole	Alkaloids	Plumerane
MWSmce349	Laurocapram	Alkaloids	Alkaloids
MWSmce448	Imidazol-1-yl-acetic acid	Alkaloids	Alkaloids
MWSslk149	5-Methoxysalicylic acid	Phenolic acids	Phenolic acids
MWStz202	cis-Moschamine	Alkaloids	Plumerane
MWStz282	3-Hydroxy-3-acetonyloxindole	Alkaloids	Plumerane
NK10264324	Phloroglucinol; 1,3,5-Benzenetriol	Phenolic acids	Phenolic acids
pma0149	Sinapoyl malate	Phenolic acids	Phenolic acids
pmb0530	Nicotinic acid adenine dinucleotide	Nucleotides and derivatives	Nucleotides and derivatives
pmb0770	N-Feruloylserotonin	Alkaloids	Plumerane
pmb0786	D-Glucosamine	Others	Saccharides
pmb0855	LysoPC 16:0	Lipids	LPC
pmb0876	LysoPE 16:0	Lipids	LPE
pmb1466	Tricin-4'-O-syringic acid	Flavonoids	Flavones
pmb1754	O-Phosphocholine	Alkaloids	Alkaloids
pmd0132	LysoPC 16:0(2n isomer)	Lipids	LPC
pmd0136	LysoPC 18:0(2n isomer)	Lipids	LPC
pme0014	L-Glutamic acid	Amino acids and derivatives	Amino acids and derivatives
pme0075	N-Acetyl-L-glutamic acid	Amino acids and derivatives	Amino acids and derivatives

pme0122	N6-Acetyl-L-lysine	Amino acids and derivatives	Amino acids and derivatives
pme0278	2,6-Diaminooimelic acid	Organic acids	Organic acids
pme1474	5'-Deoxy-5'-(methylthio)adenosine	Nucleotides and derivatives	Nucleotides and derivatives
pme1611	Isohemiphloin	Flavonoids	Flavanones
pme2060	N6-Isopentenyladenine	Nucleotides and derivatives	Nucleotides and derivatives
pme2063	Cis-Zeatin	Alkaloids	Alkaloids
pme2954	Quercetin	Flavonoids	Flavonols
pme3096	Aminomalonic acid	Organic acids	Organic acids
pme3382	N-Acetyl-L-threonine	Amino acids and derivatives	Amino acids and derivatives
pme3459	Cafestol	Terpenoids	Ditepenoids
pmf0297	1-Eicosanol	Lipids	Free fatty acids
pmn001606	Eicosenoic acid	Lipids	Free fatty acids
pmn001694	9,10,13-Trihydroxy-11-Octadecenoic Acid	Lipids	Free fatty acids
pmp000004	4',5,7-Trihydroxy-3',6-dimethoxyflavone (Jaceosidin)	Flavonoids	Flavones
pmp001251	LysoPC 18:2(2n isomer)	Lipids	LPC
pmp001264	Hexadecylsphingosine	Lipids	Sphingolipids
pmp001273	LysoPC 18:2	Lipids	LPC
pmp001281	LysoPC 18:1	Lipids	LPC
Qmxp082605	Sculponeatin A	Terpenoids	Ditepenoids
Qmyp101229	1β-Hydroxy-α-cyperone	Terpenoids	Sesquiterpenoids
Qmzp101908	5'-Methoxylariciresinol	Lignans and Coumarins	Lignans

Waln011222	2-(2,3-dihydroxypropoxy)-3-(((2-(dimethylamino)ethoxy)(hydroxy)phosphoryl)oxy)propyl palmitate	Lipids	LPE
Waln011524	1-(2,3-dihydroxypropoxy)-3-(((2-(dimethylamino)ethoxy)(hydroxy)phosphoryl)oxy)propan-2-yl palmitate	Lipids	LPE
Waln013216	10-Hydroxystearic Acid	Lipids	Free fatty acids
Walp006011	2-Aminotetradecane-1,4-diol	Lipids	Sphingolipids
Walp007043	2-Aminohexadecane-1,4-diol	Lipids	Sphingolipids
Walp007190	2-AminoicoSane-1,5,7,19-tetraol	Lipids	Sphingolipids
Wbsp000219	Apigenin-4'-O-(2",6"-di-O-p-coumaroyl)glucoside	Flavonoids	Flavones
Wbsp007290	Lycoposerramine E	Alkaloids	Alkaloids
Wbtp008378	1,3,5,6-tetrahydroxy-2-(3-methylbut-2-en-1-yl)xanthen-9-one	Flavonoids	Other Flavonoids
Wccp011838	Octadec-8-enamide	Alkaloids	Alkaloids
Wcgp005256	(3R)-3-hydroxy-6-(2-hydroxyethyl)-7-(hydroxymethyl)-5-methyl-2-methylidene-3H-inden-1-one	Others	Others
Wcsn010349	Moniliferanone D	Others	Others
Wcsn011125	Ethyl (4E,6E,8E,10E,13Z)-15,16-dihydroxy-5,9-dimethyloctadeca-4,6,8,10,13-pentaenoate	Others	Others
Wmzn000627	Ajugalaevigatic acid	Terpenoids	Ditepenoids
Xmzn007677	Ergotamine	Alkaloids	Plumerane
Zaxn005611	Methyl abieta	Terpenoids	Ditepenoids
Zaxn005652	Pisiferic acid	Terpenoids	Ditepenoids

Zaxn005783	Methyl neoabietate	Terpenoids	Ditepenoids
Zbsp010454	[4]-Shogaol dimethyl	Phenolic acids	Phenolic acids
ZINC14881154	9-Hexadecenoic acid	Lipids	Free fatty acids
zjbp110818	6,7-Dimethoxy-2-(2-phenylethyl)chromone	Others	Chromone
Zmbp002461	3-(2'-Hydroxyl-phenyl)-4-(3H)-quinazolone	Alkaloids	Alkaloids
Zmcp008957	Spirost-5-en-3,17,24-triol (24-Hydroxypennogenin)	Steroids	Steroidal saponins
Zmcp102206	(Z)-3,8-Dihydro 6,6·;7,3'a-diligustilide	Others	Others
Zmdn001564	γ-Glutamylphenylalanine	Amino acids and derivatives	Amino acids and derivatives
Zmdn004052	Aloe emodin-1-O-glucoside	Quinones	Anthraquinone
Zmdp001647	γ-Glutamyl-L-valine	Amino acids and derivatives	Amino acids and derivatives
Zmdp002216	L-γ-Glutamyl-L-leucine	Amino acids and derivatives	Amino acids and derivatives
Zmgn000217	Itaconic acid	Organic acids	Organic acids
Zmgp003228	5-hydroxy-1-phenyl-7-(3,4-dihydroxyphenyl)-3-heptanone	Others	Ketone compounds
Zmhp003514	6,7,8-Tetrahydroxy-5-methoxyflavone*	Flavonoids	Flavones
Zmmp003443	Bestim	Amino acids and derivatives	Amino acids and derivatives
Zmpn000199	D-Galactaric acid	Others	Saccharides
Zmxn004029	7,8-Dihydro-Buddlenol B(erythro)	Phenolic acids	Phenolic acids
Zmyn005015	Palmitoylethanolamide	Lipids	Free fatty acids

Zmzn000113	L-threo-3-Methylaspartate	Amino acids and derivatives	Amino acids and derivatives
Zmzp000145	Trimethyllysine	Amino acids and derivatives	Amino acids and derivatives

Module Colors: grey

Index	Compounds	Class I	Class II
Cmsp005051	Isolariciresinol-9-O-glucoside	Lignans and Coumarins	Lignans
Cmzn005251	6-O-Caffeoylarbutin	Phenolic acids	Phenolic acids
HJN093	2-O-Salicyl-6-O-Galloyl-D-Glucose	Phenolic acids	Phenolic acids
Hmbp001276	Gallacetophenone	Phenolic acids	Phenolic acids
Hmcp009963	Candicine	Alkaloids	Phenolamine
Hmgp003086	Tricin-7-O-rutinoside*	Flavonoids	Flavones
Hmqn003054	9,10,11-Trihydroxy-12-octadecenoic acid	Lipids	Free fatty acids
Ladn005758	Graminone B	Lignans and Coumarins	Lignans
Ladp007948	Oleracone B	Others	Ketone compounds
Lamp000484	4-Methylazetidine-2-Carboxylic acid*	Organic acids	Organic acids
Lasp003640	2,4-Dihydroxy-6-methoxyacetophenone	Others	Ketone compounds
Lazn001573	Erythro-Guaiacylglycerol	Phenolic acids	Phenolic acids
Lazn002744	C-Veratroylglycol	Phenolic acids	Phenolic acids
Lcyp000022	26-Desglucoavenacoside A	Terpenoids	Triterpene Saponin
Lhhp005648	Trans-3,4-methylenedioxycinnamyl alcohol*	Lignans and Coumarins	Coumarins
Lmcn009122	(7Z)-Hexadecenoic acid*	Lipids	Free fatty acids
Lmcp000282	L-Carnitine	Alkaloids	Alkaloids
Lmcp007240	5,6,7,4'-Tetramethoxyflavone	Flavonoids	Flavones
Lmgp004152	Diosmetin-8-C-(2''-O-arabinosyl)glucoside	Flavonoids	Flavones
Lmgp004959	Tricin-7-O-neohesperidoside*	Flavonoids	Flavones
Lmhn003240	Benzoylmalic acid	Phenolic acids	Phenolic acids
Lmhp001732	L-Prolyl-L-Phenylalanine	Amino acids and derivatives	Amino acids and derivatives

Lmjp004533	Kisasagenol A Triacetate	Terpenoids	Terpene
Lmjp101403	3ξ-(1ξ-Hydroxyethyl)-7-hydroxy-1-isobenzofuranone	Others	Others
Lmlp001436	Dihydrocaffeic acid	Phenolic acids	Phenolic acids
Lmlp004754	Ehretioside B	Alkaloids	Phenolamine
Lmmn006306	Machilusolide D	Others	Lactones
Lmmp002080	N-(4-Aminobutyl)benzamide	Alkaloids	Alkaloids
Lmnp102580	Apigenin-6-C-(2''-xylosyl)glucoside*	Flavonoids	Flavones
Lmnp202682	5,7,4'-Trihydroxy-8-methoxyflavone-6-C-[Xylosyl-(1-2)]-glucoside	Flavonoids	Flavones
Lmpn007255	Patuletin (Quercetagetin-6-methyl ether)	Flavonoids	Flavonols
Lmqp000427	N-Methyl-Trans-4-Hydroxy-L-Proline	Amino acids and derivatives	Amino acids and derivatives
Lmsn004674	1-O-Cinnamoyl-β-D-glucose	Phenolic acids	Phenolic acids
Lmtp002642	Apigenin-8-C-(2''-glucosyl)arabinoside	Flavonoids	Flavones
Lmtp002942	Apigenin-6,8-di-C-arabinoside*	Flavonoids	Flavones
Lmyp003025	Coixlactam	Alkaloids	Quinoline alkaloids
Lskp211385	(-)-dihydrosesamin*	Lignans and Coumarins	Lignans
Lskp211426	DihydroStigmast-4-ene-3,6-dione	Steroids	Steroid
MEDP1785	Methyl vanillate	Phenolic acids	Phenolic acids
ML10181668	Cycloleucine	Amino acids and derivatives	Amino acids and derivatives
ML10195036	3-Dehydrosphinganine	Lipids	Sphingolipids
MW0012723	19,20-DiHDPA	Lipids	Free fatty acids
MW0014493	4-Hydroxycrotonic acid	Organic acids	Organic acids
MW0015494	15-keto-PGF1alpha	Lipids	Free fatty acids
MW0017122	Ciprostene	Organic acids	Organic acids

MW0106526	3-phenyl-2-(pyrrolidine-2-carbonylamino)propanoic Acid	Amino acids and derivatives	Amino acids and derivatives
MW0108648	Ethyl N-acetyl-L-tyrosinate	Amino acids and derivatives	Amino acids and derivatives
MW0110454	Val-Tyr	Amino acids and derivatives	Amino acids and derivatives
MW0158880	Val-Ala-Ser-Asp	Amino acids and derivatives	Amino acids and derivatives
MW0159001	Val-Glu-Trp	Amino acids and derivatives	Amino acids and derivatives
mws0009	Coniferaldehyde*	Phenolic acids	Phenolic acids
mws0014	Ferulic acid*	Phenolic acids	Phenolic acids
mws0017	Spermidine	Alkaloids	Alkaloids
MWS00330g	1-Amino-1-cyclobutane-carboxylic-acid*	Amino acids and derivatives	Amino acids and derivatives
mws0055	Tangeretin (4',5,6,7,8-Pentamethoxyflavone)	Flavonoids	Flavones
mws0216	Trans-4-Hydroxy-L-proline	Amino acids and derivatives	Amino acids and derivatives
mws0256	L-Valine	Amino acids and derivatives	Amino acids and derivatives
mws0258	L-Isoleucine*	Amino acids and derivatives	Amino acids and derivatives
mws0361	Palmitoleic Acid*	Lipids	Free fatty acids
mws0362	Heptadecanoic acid	Lipids	Free fatty acids
mws0371	Cis-4,7,10,13,16,19-Docosahexaenoic Acid	Lipids	Free fatty acids
mws0376	Fumaric acid	Organic acids	Organic acids

MWS0442	Maltotriose	Others	Saccharides
mws0444	3-Aminosalicylic acid	Phenolic acids	Phenolic acids
MWS0550	Cinnamaldehyde	Phenolic acids	Phenolic acids
MWS0552	Cis-10-Pentadecenoic Acid(C15:1)	Lipids	Free fatty acids
mws0628	4-Hydroxybenzaldehyde	Phenolic acids	Phenolic acids
mws0677	N-Acetyl-5-hydroxytryptamine	Alkaloids	Plumerane
mws0889	D-Threonic Acid	Others	Saccharides
mws0921	p-Coumaryl alcohol	Phenolic acids	Phenolic acids
mws0983	N-Oleoylethanolamine	Alkaloids	Alkaloids
mws1073	Apigenin-6,8-di-C-glucoside (Vicenin-2)	Flavonoids	Flavones
mws1138	Betanin (Betanidin-5-O-glucoside)	Alkaloids	Plumerane
mws1200	p-Coumaric acid methyl ester	Phenolic acids	Phenolic acids
mws1290	Kaempferol-3-O-(6"-p-Coumaroyl)glucoside (Tiliroside)	Flavonoids	Flavonols
mws1349	Dihydrosphingosine	Lipids	Sphingolipids
mws1350	Syringaldehyde; 4-Hydroxy-3,5-Dimethoxybenzaldehyde	Phenolic acids	Phenolic acids
MWS201398	Hyp-Ser	Amino acids and derivatives	Amino acids and derivatives
MWS201478	Phe-Ser	Amino acids and derivatives	Amino acids and derivatives
MWS201479	Tyr-Gly	Amino acids and derivatives	Amino acids and derivatives
mws2104	D-Pinitol	Others	Saccharides
mws2608	N-Acetyl-D-galactosamine	Others	Saccharides
MWS3035	N-Methyl-L-Glutamate	Amino acids and derivatives	Amino acids and derivatives

MWS4285	L-Lactic Acid	Organic acids	Organic acids
MWS4309	Glycyl-tryptophan	Amino acids and derivatives	Amino acids and derivatives
mws5035	L-Leucyl-L-phenylalanine	Amino acids and derivatives	Amino acids and derivatives
MWSHC20100	lycopodone*	Flavonoids	Flavones
MWSHY0018	Nobiletin (5,6,7,8,3',4'-Hexamethoxyflavone)	Flavonoids	Flavones
MWSmce039	Isonicotinic acid	Others	Vitamin
MWSmce341	Butyl isobutyl phthalate	Phenolic acids	Phenolic acids
MWSmce587	2,6-Dimethoxybenzoic acid	Phenolic acids	Phenolic acids
MWSmce607	DL-Threonine	Amino acids and derivatives	Amino acids and derivatives
MWSmce663	Vanillyl alcohol	Phenolic acids	Phenolic acids
MWSmce689	Methyl linolenate	Lipids	Free fatty acids
MWSmce706	Cyclo(Ala-Gly)	Amino acids and derivatives	Amino acids and derivatives
MWSslk208	Kaurenoic Acid	Terpenoids	Ditepenoids
MWStz091	Cyclo(L-Ala-L-Pro)	Amino acids and derivatives	Amino acids and derivatives
pma3649	5-Aminolevulinic Acid	Alkaloids	Alkaloids
pma6218	O-MethylNaringenin-8-C-arabinoside	Flavonoids	Flavanones
pmb0865	LysoPC 18:3(2n isomer)	Lipids	LPC
pmb0952	Thiamine (Vitamin B1)	Others	Vitamin
pmb1650	Octadeca-11E,13E,15Z-trienoic acid	Lipids	Free fatty acids
pme0281	Terephthalic acid	Phenolic acids	Phenolic acids

pme0282	Phthalic acid	Phenolic acids	Phenolic acids
pme0422	Isoferulic Acid*	Phenolic acids	Phenolic acids
pme0490	Nicotinic acid (Vitamin B3)*	Others	Vitamin
pme1137	6-Hydroxynicotinic acid	Alkaloids	Pyridine alkaloids
pme1419	L-Methionine methyl ester	Amino acids and derivatives	Amino acids and derivatives
pme1665	Isovitexin-7-O-glucoside(Saponarin)	Flavonoids	Flavones
pme2134	D-Threitol	Others	Saccharides
pme2237	Dulcitol*	Others	Saccharides
pme2268	Trigonelline	Alkaloids	Pyridine alkaloids
pme2292	Putrescine	Alkaloids	Alkaloids
pme3009	Trans-Citridic acid	Organic acids	Organic acids
pme3388	Homoarginine	Amino acids and derivatives	Amino acids and derivatives
pme3437	Eudesmic acid (3,4,5-trimethoxybenzoic acid)	Phenolic acids	Phenolic acids
pmf0096	Oxalic acid	Organic acids	Organic acids
pmp000452	Annuionone D	Others	Ketone compounds
pmp001106	Vitexin-2''-O-glucoside	Flavonoids	Flavones
pmp001287	N-Benzylmethylene isomethylamine	Alkaloids	Alkaloids
Wbmn013660	3,9-Dihydroxy-13(14)-labden-16,15-olide	Terpenoids	Ditepenoids
Wbmp002283	A-hydroxyquinoline	Alkaloids	Alkaloids
Wbtn007603	9,11-dimethoxy-2h-[1,3]dioxolo[4,5-b]xanthen-10-one*	Flavonoids	Other Flavonoids
Wbwn006953	Cagayanone A	Lignans and Coumarins	Lignans
Wbwp006397	(3r,4r)-3-(2h-1,3-benzodioxol-5-ylmethyl)-4-[(4-hydroxy-3-methoxyphenyl)methyl]oxolan-2-one	Lignans and Coumarins	Lignans
Wbwp008548	[(1R,2S)-1-(1,3-benzodioxol-5-yl)-2-methyl-3-oxobutyl]4-hydroxy-3-methoxybenzoate*	Lignans and Coumarins	Lignans

Wcjp002598	8-hydroxyquinoline	Alkaloids	Quinoline alkaloids
Wcsn009636	Mediterraneone	Others	Others
Wcsn011371	1-Stearidonoyl-Glycerol	Lipids	Glycerol ester
Ymjm000062	3-O-methyl furanovibsanin	Terpenoids	Triterpene
Yshs000041	Vibsanin J	Terpenoids	Ditepenoids
Zajp007311	3',4',5',5,7-Pentamethoxyflavone*	Flavonoids	Flavones
zjbp110804	6,7-dimethoxy-2-[2-(4'-hydroxy-3'-methoxyphenyl)ethyl]chromone*	Others	Chromone
Zmcp102201	Senkyunolide B*	Others	Lactones
Zmdp000972	S-Methyl-L-cysteine	Amino acids and derivatives	Amino acids and derivatives
Zmjp003031	Orientin-2''-O-galactoside	Flavonoids	Flavones
Zmjp013616	12,13-Dihydroursolic acid	Terpenoids	Triterpene
Zmpp001726	Epinephrine	Alkaloids	Alkaloids
Zmyn004449	9-Hydroxy-12-oxo-10(E),15(Z)-octadecadienoic acid	Lipids	Free fatty acids
Zmyn005384	2R-Hydroxyoctadecanoic Acid*	Lipids	Free fatty acids
Zmzp005934	Stearamide	Alkaloids	Alkaloids

Module Colors: turquoise

Index	Compounds	Class I	Class II
Cmbp005948	1-O-p-Hydroxycinnamoyl-3-O-caffeoylglycerol	Phenolic acids	Phenolic acids
Cmdn000784	Uric acid	Nucleotides and derivatives	Nucleotides and derivatives
Cmdn004031	R-Campneoside II	Phenolic acids	Phenolic acids
Cmhp007046	Cauloside D	Terpenoids	Triterpene Saponin
Cmhp007835	Cauloside A	Terpenoids	Triterpene Saponin
Cmmp006152	Norarjunolic acid	Terpenoids	Triterpene
Cmpp003619	6,7-Dihydroxy-4-methylcoumarin	Lignans and Coumarins	Coumarins
Cmsn006847	9,10-Dihydro-2-methoxy-4,5-phenanthrenediol	Quinones	PhenAnthraquinones
Cmxp005606	13-Deoxychilenine	Alkaloids	Isoquinoline alkaloids
Cmyp003227	Tetrahydroprotopapaverine	Alkaloids	Isoquinoline alkaloids
Cmyp004430	Dehydroyanhunine	Alkaloids	Isoquinoline alkaloids
Cmyp004481	Yanhusuine	Alkaloids	Isoquinoline alkaloids
Hajp007218	5,7,3',5'-tetrahydroxy-6-methylfavanone*	Flavonoids	Flavones
Hamp001749	pycnarrhine	Alkaloids	Alkaloids
Hamp003839	Griffithdione	Alkaloids	Alkaloids
Hayp001837	arenarine A	Alkaloids	Alkaloids
HJAP050	N-Feruloyl-3-methoxytyramine	Alkaloids	Phenolamine
HJN012	4,7,9,9'-Tetrahydroxy-3,3'-dimethoxy-8-O-4'-neolignan	Phenolic acids	Phenolic acids
HJN025	4-Hydroxybenzyl Alcohol	Phenolic acids	Phenolic acids
HJN037	4-O-Methylgallic Acid	Phenolic acids	Phenolic acids
HJN056	Sesquimarocanol B	Phenolic acids	Phenolic acids
HJN106	2-Carboxy-3-hydroxy-A(1)-norlupan-20(29)-en-28-oic acid (Ceanothic acid)	Terpenoids	Triterpene
Hmbn002692	6'-O-Feruloyl-D-sucrose	Phenolic acids	Phenolic acids

Hmbn004734	3,23-Dihydroxy-30-noroleana-12,20(29)-dien-28-oic acid (30-Norhederagenin)	Terpenoids	Triterpene
Hmbp006861	1,2-O-Diferuloylglycerol	Phenolic acids	Phenolic acids
Hmcn000192	Sedoheptulose	Others	Saccharides
Hmcn002352	Ailantinol F	Terpenoids	Ditepenoids
Hmcn002743	Lirioresinol A	Lignans and Coumarins	Lignans
Hmcp003852	Elemol	Terpenoids	Sesquiterpenoids
Hmcp006520	1,3-Dihydroxypropan-2-yl 12-methyl-6-(6-methylheptyl)tridec-12-enoate	Others	Others
Hmdp003220	Corytuberine	Alkaloids	Aporphine alkaloids
Hmdp005429	Jatrorrhizine	Alkaloids	Isoquinoline alkaloids
Hmfn000531	L-Ascorbic acid (Vitamin C)	Others	Vitamin
Hmgn002833	4-Ketopinoresinol	Lignans and Coumarins	Lignans
Hmgn004693	8-Hydroxy-α-conidendrin	Lignans and Coumarins	Lignans
Hmgp002121	Hispidulin-7-O-(6''-O-p-Coumaroyl)Glucoside	Flavonoids	Flavones
Hmhp001812	2'-O-Methyladenosine	Nucleotides and derivatives	Nucleotides and derivatives
Hmhp006831	Kokusaginine	Alkaloids	Quinoline alkaloids
Hmjn003948	2,3,6-Trihydroxyurs-12-en-28-oic acid (Madasiatic acid)	Terpenoids	Triterpene
Hmjn008136	2,3-Dihydroxyoleana-11,13(18)-dien-28-oic acid (Camaldulenic acid)	Terpenoids	Triterpene
Hmjp005089	(2Z)-N-[2-(3,4-Dihydroxyphenyl)-2-hydroxyethyl]-3-(4-methoxyphenyl)-2-propenamide	Alkaloids	Phenolamine
Hmjp005556	(2E)-3-(4-Hydroxyphenyl)-N-[2-(4-hydroxyphenyl)ethyl]-2-propenamide	Alkaloids	Alkaloids
Hmln000297	Inositol*	Others	Saccharides
Hmlp000935	Vanillylamine	Alkaloids	Phenolamine
Hmlp003129	Matairesinol	Lignans and Coumarins	Lignans
Hmlp006688	durupcoside C	Terpenoids	Triterpene Saponin
Hmmp005965	Saponin PC	Terpenoids	Triterpene Saponin
Hmmp007438	Mimengoside F	Terpenoids	Triterpene Saponin

Hmqn002118	7-Hydroxycoumarin;Umbelliferone	Lignans and Coumarins	Coumarins
Hmsn002598	Salirepin	Phenolic acids	Phenolic acids
Hmsp000364	L-Cyclopentylglycine	Amino acids and derivatives	Amino acids and derivatives
Hmsp003093	3,7-Dihydroxychromen-4-one	Others	Chromone
Hmtn001120	5-(2-Hydroxyethyl)-2-O-glucosylphenol*	Phenolic acids	Phenolic acids
Hmtn001288	Methyl 2,4-dihydroxyphenylacetate*	Phenolic acids	Phenolic acids
HX1191	3,4-Secodammara-4(28),20,24-trien-3,26-dioic acid	Terpenoids	Triterpene
HX1208	Cristacarpin	Lignans and Coumarins	Lignans
HX1226	4-Hydroxy-β-bulnesene	Others	Others
HX1348	2-hydroxymethyl-D-ribono-γ-lactone	Others	Others
HX1349	dihydro-4-hydroxy-5-hydroxymethyl-2(3H)-furanone	Others	Others
HX1362	aesculetine	Lignans and Coumarins	Coumarins
HX1421	Haplopine	Alkaloids	Quinoline alkaloids
Jmbn003202	Butyl Beta-D-Fructopyranoside	Others	Saccharides
Jmhp002680	Norlotusine	Alkaloids	Isoquinoline alkaloids
Jmhp002906	N-Methylhigenamine	Alkaloids	Isoquinoline alkaloids
Jmzn006005	3,4-Methylenedioxy cinnamyl alcohol	Lignans and Coumarins	Lignans
Lacp002720	Govadine	Alkaloids	Isoquinoline alkaloids
Ladn005852	Graminone A	Lignans and Coumarins	Lignans
Lafp006517	Arctigenin	Lignans and Coumarins	Lignans
Lahp003747	Orijanone*	Alkaloids	Quinoline alkaloids
Lakn004098	Dihydrophaseic Acid	Terpenoids	Sesquiterpenoids
Lasp006564	5'-Demethylaquillochin	Lignans and Coumarins	Lignans
Latp011219	3,5-Dimethyl-2,3-dihydrobenzofuran	Others	Others

Layp002880	5,7-Dihydroxychromone glucoside	Others	Ketone compounds
Layp008215	prosapogenin B	Steroids	Steroidal saponins
Lazn002213	1-(4-Hydroxy-3-methoxyphenyl)-2-[4-(1,2,3-trihydroxypropyl)-2-methoxyphenoxy]-1,3-propanediol	Lignans and Coumarins	Lignans
Lazn002600	Erythro-Guaiacylglycerol-β-threo-syringylglycerol Ether	Lignans and Coumarins	Lignans
Lazn002951	Guaiacylglycerol-β-Guaiacyl Ether	Lignans and Coumarins	Lignans
Lazn003560	Methoxy-erythro-Guaiacyl glycerol β-threo-syringyl glycerol ether	Lignans and Coumarins	Lignans
Lazn003893	Erythro-Guaiacylglycerol-β-Coniferyl Ether	Lignans and Coumarins	Lignans
Lazn005175	1-(4-Hydroxy-3,5-dimethoxyphenyl)-2-[4-(3-hydroxypropyl)-2,6-dimethoxyphenoxy]propane-1,3-diol	Lignans and Coumarins	Lignans
Lazn005262	Erythro-Guaiacylglycerol-β-Sinapyl Ether	Lignans and Coumarins	Lignans
Lazn005628	Hedyotisol B	Lignans and Coumarins	Lignans
Lazn006616	Buddlenol F	Lignans and Coumarins	Lignans
Lazn006735	Erythro-Guaiacylglycerol-β-O-4'-dehydrodisinapyl Ether	Lignans and Coumarins	Lignans
Lazp005033	Divanillyltetrahydrofuran	Lignans and Coumarins	Lignans
Lbhp011921	Fraxetin glucuronide	Lignans and Coumarins	Coumarins
Lcgn000234	davidioside C	Phenolic acids	Phenolic acids
Lchp000008	Simulansine	Alkaloids	Alkaloids
Lchp000218	(R)-3-ethyl-7-hydroxy-6-methoxyphthalide	Others	Others
Lcrp000382	tetrahydrofuroguaiacin B	Lignans and Coumarins	Lignans
Lcsp000669	Hovenic acid	Terpenoids	Triterpene
Lcyn000686	Prunustomentosanan A	Others	Others
Lcyp000028	Avenanthramide A	Alkaloids	Phenolamine
Lcyp000042	(E)-4,5-dihydroxy-2-(3-(4-hydroxy-3-methoxyphenyl)acrylamido)benzoic acid	Alkaloids	Alkaloids
Lhbp073003	Magnocurarine	Alkaloids	Isoquinoline alkaloids
Lhbp073013	Corytenchine(tetrahydropalmatrubine)	Alkaloids	Isoquinoline alkaloids
Lhcp102418	Saikosaponin A	Terpenoids	Triterpene Saponin

Lhcp102420	Saikosaponin B2	Terpenoids	Triterpene Saponin
Lhhp102922	Fargesin	Lignans and Coumarins	Lignans
Lhhp102923	(7'R,8'R)-7'8'-dihydro-7'-(5'-hydroxy-3'-methoxphenyl)-3-methoxy-8'-methyl-1-(E)-propenylbenzof-uran	Lignans and Coumarins	Lignans
Lhhp120816	Hydroxyeugenol	Others	Others
Lhjp120709	Dimethyllimettin	Lignans and Coumarins	Coumarins
Lhjp120712	Hydroxydaphnoretin glucoside	Lignans and Coumarins	Coumarins
Lhmp101307	α-Hydroxyl-β,β-Dimethyl-γ-Butyrolactone	Others	Others
Lhmp102634	Ilexolide A	Terpenoids	Triterpene Saponin
Lhnp080309	Dehydronantenine	Alkaloids	Aporphine alkaloids
Lhnp110101	3,20-Dihydroxyurs-21-en-28-oic acid (Oleanderic acid)	Terpenoids	Triterpene
Lhwp110301	19-Hydroxy-2-(hydroxymethyl)-A(1)-norursa-2,12-dien-28-oic acid (Coleonolic acid)	Terpenoids	Triterpene
Lman002117	Grevilloside Q	Others	Ketone compounds
Lman002731	Grevilloside F	Phenolic acids	Phenolic acids
Lmap001823	Dhurrin	Alkaloids	Phenolamine
Lmbn001162	Esculetin-7-O-glucoside*	Lignans and Coumarins	Coumarins
Lmbn001181	Fraxetin-7,8-di-O-glucoside	Lignans and Coumarins	Coumarins
Lmbn002072	2-Propylsuccinic acid*	Organic acids	Organic acids
Lmbn003970	9,12,13-TriHOME; 9(S),12(S),13(S)-Trihydroxy-10(E)-octadecenoic acid	Lipids	Free fatty acids
Lmbn004790	Methyl 3-(3-hydroxy-4-methoxyphenyl)propanoate*	Phenolic acids	Phenolic acids
Lmbn004847	4-Methoxyphenylpropionic acid	Phenolic acids	Phenolic acids
Lmbn005287	7S,8S-DiHODE; (9Z,12Z)-(7S,8S)-Dihydroxyoctadeca-9,12-dienoic acid*	Lipids	Free fatty acids
Lmbn005487	12,13-DHOME; (9Z)-12,13-Dihydroxyoctadec-9-enoic acid	Lipids	Free fatty acids
Lmbn014696	Pimaric acid	Terpenoids	Ditepenoids
Lmbp000123	L-Homomethionine	Amino acids and derivatives	Amino acids and derivatives

Lmbp001216	L-Dihomomethionine	Amino acids and derivatives	Amino acids and derivatives
Lmbp002672	8,14-Dihydroflavinantine	Alkaloids	Isoquinoline alkaloids
Lmbp002962	Isofraxetin	Lignans and Coumarins	Coumarins
Lmbp003825	Skimmin (7-Hydroxycoumarin-7-O-glucoside)	Lignans and Coumarins	Coumarins
Lmcn004052	Buddlenol D	Phenolic acids	Phenolic acids
Lmcp000611	2-Amino-4,5-dihydro-1H-imidazole-4-acetic acid	Alkaloids	Alkaloids
Lmcp004369	Quercetin-3',4'-dimethyl ether	Flavonoids	Flavonols
Lmdn006811	3,24-Dihydroxyolean-12-en-22-one (Soyasapogenol E)	Terpenoids	Triterpene
Lmdn009099	Coniferyl ferulate	Phenolic acids	Phenolic acids
Lmfn006411	Cryptostrobin (8-C-Methyl-5,7-Dihydroxyflavanone)	Flavonoids	Flavanones
Lmgn000188	D-Lactic Acid	Organic acids	Organic acids
Lmgn001670	Salicylic acid	Phenolic acids	Phenolic acids
Lmgp002593	3-hydroxybenzaldehyde	Phenolic acids	Phenolic acids
Lmhn002140	Feruloyltartaric acid (Fertaric acid)	Phenolic acids	Phenolic acids
Lmhn002437	Terreic acid	Others	Ketone compounds
Lmhp001461	L-Prolyl-L-Leucine	Amino acids and derivatives	Amino acids and derivatives
Lmhp001670	L-Valyl-L-Leucine	Amino acids and derivatives	Amino acids and derivatives
Lmhp002001	L-Valyl-L-Phenylalanine	Amino acids and derivatives	Amino acids and derivatives
Lmhp206353	Tricin-4'-O-glucoside*	Flavonoids	Flavones
Lmjp004941	3,5,4'-Trihydroxy-7-methoxyflavone (Rhamnocitrin)*	Flavonoids	Flavonols
Lmlp001567	4'-Hydroxy-6,7-dimethoxyl-N,N-dimethyltetrahydroisoquinoline	Alkaloids	Isoquinoline alkaloids

Lmlp002990	Isosaponarin(Isovitexin-4'-O-glucoside)	Flavonoids	Flavones
Lmlp002994	5-Hydroxyquinoline	Alkaloids	Quinoline alkaloids
Lmmn000774	Dihydrocaffeoylglucose	Phenolic acids	Phenolic acids
Lmmn000806	Dimethylmalonic acid*	Organic acids	Organic acids
Lmmn002179	Methyl salicylate-2-O-glucoside	Phenolic acids	Phenolic acids
Lmmn003323	2-Hydroxyhexadecanoic acid	Organic acids	Organic acids
Lmmn003748	Isolariciresinol	Lignans and Coumarins	Lignans
Lmnn004130	Lappaol C	Lignans and Coumarins	Lignans
Lmnp202580	Apigenin-8-C-(2''-xylosyl)glucoside*	Flavonoids	Flavones
Lmqn001795	5-Hydroxy-7-methoxyflavone	Flavonoids	Flavones
Lmqp002330	Sinomendine	Alkaloids	Aporphine alkaloids
Lmqp002340	13,13α-Didehydro-9,10-dimethoxy-2,3-(methylenedioxy)-berbine	Alkaloids	Isoquinoline alkaloids
Lmqp003432	6,9-Dihydroxy-7-megastigmen-3-one	Terpenoids	Sesquiterpenoids
Lmrj001341	Cyclo(Ser-Pro)	Amino acids and derivatives	Amino acids and derivatives
Lmrj002087	L-Isoleucyl-L-Aspartate	Amino acids and derivatives	Amino acids and derivatives
Lmrj002244	Cyclo(Pro-Pro)	Amino acids and derivatives	Amino acids and derivatives
Lmsn000954	Dambonitol	Others	Saccharides
Lmsn011830	2,19-Dihydroxy-3-oxours-12-en-28-oic acid	Terpenoids	Triterpene
Lmsp004450	Dehydrodiconiferyl alcohol	Lignans and Coumarins	Lignans
Lmsp008443	3,3',5-Trihydroxy-4',7-dimethoxyflavanone	Flavonoids	Flavanones
Lmsp013116	Ent-16α,17-Dihydroxykauran-2-one	Terpenoids	Ditepenoids
Lmtn002796	Aromadendrin-7-O-glucoside*	Flavonoids	Flavanonols

Lmtn004049	Abscisic acid	Organic acids	Organic acids
Lmtp002822	Apigenin-6-C-arabinoside-8-C-xyloside*	Flavonoids	Flavones
Lmwn102817	Glaucin A	Steroids	Steroid
Lmwn102819	Rutaevin acetate	Steroids	Steroid
Lmwn102907	Evodol	Terpenoids	Triterpene
Lmwp102713	(S)-Peucedanol	Lignans and Coumarins	Coumarins
Lmxp001668	2-Methoxy-4-ethenylphenol	Phenolic acids	Phenolic acids
Lmyn000160	Mucic acid Dimethyl Ester	Phenolic acids	Phenolic acids
Lmyn002788	Methyl gallate*	Phenolic acids	Phenolic acids
Lmyn011818	3,19-Epoxy-3,22-dihydroxydammara-20,24-dien-26-oic acid δ-lactone (Semialactone)	Terpenoids	Triterpene
Lmyn012771	3-Oxoolean-18-en-28-oic acid (Moronic acid)	Terpenoids	Triterpene
Lmzn006024	2-Aldehydo-A(1)-norlup-20(29)-en-27,28-dioic acid (Zizyberanal acid)	Terpenoids	Triterpene
Lmzn006795	3-Oxours-12-en-28-oic acid (Ursonic acid)*	Terpenoids	Triterpene
Lmzn106829	3-Oxolup-20(29)-en-28-oic acid (Betulonic acid)	Terpenoids	Triterpene
Lmzn206829	3-Oxoolean-12-en-28-oic Acid (Oleanonic acid)*	Terpenoids	Triterpene
Lmzp002365	Hesperetin-7-O-glucoside	Flavonoids	Flavanones
lsdp210009	Elaeocarpucin F	Terpenoids	Triterpene
Lshp011103	Echinulin	Alkaloids	Plumerane
Lsjp211242	N-(1-hydroxymethyl-2-phenylethyl)benzamide	Alkaloids	Alkaloids
Lsjp211249	Teuvincenone E	Terpenoids	Ditepenoids
Lsjp211302	Spirost-4-en-3-one (Diosgenone)	Steroids	Steroid
Lskp211249	epieudesmin	Lignans and Coumarins	Lignans
Lskp211268	3-Ethyl-7-hydroxyphthslide	Others	Others
Lskp211461	Olean-12-ene-3,16-dione (Maniladione)	Terpenoids	Triterpene
Lskp211484	19-Hydroxy-3-oxo-24-norolean-12-en-28-oic acid(ISO3)	Terpenoids	Triterpene

Lsmp121507	Folifine	Alkaloids	Quinoline alkaloids
Lsmp121515	Dehydroxyribalinine	Alkaloids	Quinoline alkaloids
Lssp210101	Kushenol S	Flavonoids	Other Flavonoids
Lssp210247	1,3,5,5,6-tetrahydroxy-4-isopentenyl xanthone	Others	Others
Lssp210310	3-hydroxylup-12(13),20(29)-diene	Terpenoids	Triterpene
Lssp210386	1,6-dihydroxy-5-methoxy-4',4'dimethyl-4'''5'-dihydropyT-6'-one(2',3':3,4)xanthone	Others	Others
Lsxp211202	Hydrohydrastinine	Alkaloids	Alkaloids
Lszp210010	Tatarine	Alkaloids	Alkaloids
Lwhp010601	glyconone	Alkaloids	Piperidine alkaloids
Lwhp010603	Dehydroxyevodol	Terpenoids	Terpene
Lwhp011003	Clauemargine A	Terpenoids	Terpene
MA10012327	D-Sphingosine	Lipids	Sphingolipids
MEDL00397	3-hydroxy-tetradecanoic acid	Organic acids	Organic acids
MEDL01876	(8)-Shogaol	Phenolic acids	Phenolic acids
MW0000741	4-Tert-butylphenoxyacetic acid	Organic acids	Organic acids
MW0003614	3,4-Dihydro-8-hydroxy-3-(3-hydroxy-4-methoxyphenyl) 1H-2-benzopyran-1-one	Phenolic acids	Phenolic acids
MW0009415	Phenylacetic acid	Organic acids	Organic acids
MW0012183	12S-Hepe	Lipids	Free fatty acids
MW0014140	2-Hydroxy-3-isopropylsuccinic acid	Organic acids	Organic acids
MW0014329	4-(Methylsulfanyl)-2-oxobutanoic acid	Organic acids	Organic acids
MW0015455	(10E,12Z)-9-HODE	Lipids	Free fatty acids
MW0063708	Sumaresinol	Terpenoids	Triterpene
MW0106308	Cystathionine	Amino acids and derivatives	Amino acids and derivatives

MW0107019	Glutamylproline	Amino acids and derivatives	Amino acids and derivatives
MW0107714	Leucyl-aspartyl-valine	Amino acids and derivatives	Amino acids and derivatives
MW0109421	Prolylproline	Amino acids and derivatives	Amino acids and derivatives
MW0116559	Tryptoline	Alkaloids	Plumerane
MW0118724	2-Methyl-3-(pyrimidin-2-YL)propanoic acid	Organic acids	Organic acids
MW0120912	5-(Tetradecyloxy)-2-furoic acid	Organic acids	Organic acids
MW0125173	2H-Pyran-2-one, tetrahydro-4-hydroxy-4-methyl-, (R)-	Others	Lactones
MW0127882	2,2'-Dihydroxy-4',6'-dimethoxychalcone	Others	Ketone compounds
MW0135131	4',6-Dihydroxyflavone	Flavonoids	Flavones
MW0137868	Daphnin*	Lignans and Coumarins	Coumarins
MW0144456	Ala-Ala-His	Amino acids and derivatives	Amino acids and derivatives
MW0144571	Ala-Glu-Ser-Glu	Amino acids and derivatives	Amino acids and derivatives
MW0145747	Asn-Glu-Thr-Glu	Amino acids and derivatives	Amino acids and derivatives
MW0146118	Asp-Gly-Phe	Amino acids and derivatives	Amino acids and derivatives
MW0147993	Cys-Asp-Val	Amino acids and derivatives	Amino acids and derivatives
MW0148010	Cys-Gly-Glu	Amino acids and derivatives	Amino acids and derivatives

MW0150440	Gly-Met-Tyr	Amino acids and derivatives	Amino acids and derivatives
MW0150890	His-Cys-Tyr	Amino acids and derivatives	Amino acids and derivatives
MW0151089	His-Ser-Val-Glu	Amino acids and derivatives	Amino acids and derivatives
MW0152629	H-Leu-Trp-OH	Amino acids and derivatives	Amino acids and derivatives
MW0153484	Met-Ala-Glu	Amino acids and derivatives	Amino acids and derivatives
MW0153514	Met-Asp-Glu	Amino acids and derivatives	Amino acids and derivatives
MW0153515	Met-Asp-Gly	Amino acids and derivatives	Amino acids and derivatives
MW0153668	Met-Ser-Tyr	Amino acids and derivatives	Amino acids and derivatives
MW0153739	Met-Abu-OH	Amino acids and derivatives	Amino acids and derivatives
MW0154030	13-Tetradecynoic acid	Lipids	Free fatty acids
MW0154287	Nap-Leu-OH	Amino acids and derivatives	Amino acids and derivatives
MW0154290	Nap-Nap-OH	Amino acids and derivatives	Amino acids and derivatives
MW0155819	Pro-Asp-Tyr	Amino acids and derivatives	Amino acids and derivatives

MW0155862	Pro-Glu-Val	Amino acids and derivatives	Amino acids and derivatives
MW0155972	Pro-Pro-Asp	Amino acids and derivatives	Amino acids and derivatives
MW0155982	Pro-Pro-Ser	Amino acids and derivatives	Amino acids and derivatives
MW0155984	Pro-Pro-Thr	Amino acids and derivatives	Amino acids and derivatives
MW0155992	Pro-Ser-Ile	Amino acids and derivatives	Amino acids and derivatives
MW0155996	Pro-Ser-Tyr	Amino acids and derivatives	Amino acids and derivatives
MW0156743	Ser-Glu-Ser-Asp-Ile	Amino acids and derivatives	Amino acids and derivatives
MW0156747	Ser-Glu-Tyr-Glu	Amino acids and derivatives	Amino acids and derivatives
MW0156781	Ser-His-Thr	Amino acids and derivatives	Amino acids and derivatives
MW0157598	Thr-Cys-Asp	Amino acids and derivatives	Amino acids and derivatives
MW0157677	Thr-Ile-Asp-Phe-Glu	Amino acids and derivatives	Amino acids and derivatives
MW0158269	Trp-Trp-Tyr	Amino acids and derivatives	Amino acids and derivatives
MW0158613	Tyr-Ser-Val-Glu	Amino acids and derivatives	Amino acids and derivatives

MW0159029	Val-His-Phe-Glu	Amino acids and derivatives	Amino acids and derivatives
MW0159168	Val-Ser-Val	Amino acids and derivatives	Amino acids and derivatives
MW0168579	3-Thiatetradecanoic Acid	Lipids	Free fatty acids
MW0169676	Pinolenic acid	Lipids	Free fatty acids
mws0018	Spermine	Alkaloids	Alkaloids
MWS00216g	L-Phenylalaninol	Amino acids and derivatives	Amino acids and derivatives
mws0027	Syringic acid	Phenolic acids	Phenolic acids
mws0028	Vanillic acid	Phenolic acids	Phenolic acids
mws0036	Hesperetin-7-O-rutinoside (Hesperidin)*	Flavonoids	Flavanones
mws0045	Quercetin-3-O-rhamnoside(Quercitrin)	Flavonoids	Flavonols
mws0048	Apigenin-8-C-Glucoside (Vitexin)*	Flavonoids	Flavones
mws0066	Isorhamnetin; 3'-Methoxy-3,4',5,7-Tetrahydroxyflavone	Flavonoids	Flavonols
mws0093	Coniferyl alcohol	Phenolic acids	Phenolic acids
mws0096	Piperitol	Lignans and Coumarins	Lignans
mws0097	Pinoresinol*	Lignans and Coumarins	Lignans
mws0117	Homovanillic acid; 4-Hydroxy-3-methoxyphenylacetic acid	Phenolic acids	Phenolic acids
mws0178	Chlorogenic acid (3-O-Caffeoylquinic acid)*	Phenolic acids	Phenolic acids
mws0179	Chlorogenic acid methyl ester	Phenolic acids	Phenolic acids
mws0180	2,5-Dihydroxybenzoic acid; Gentisic Acid*	Phenolic acids	Phenolic acids
mws0183	3,4-Dihydroxybenzoic acid (Protocatechuic acid)*	Phenolic acids	Phenolic acids
mws0192	Succinic acid*	Organic acids	Organic acids

mws0193	L-Homocitrulline	Amino acids and derivatives	Amino acids and derivatives
mws0208	Adipic Acid*	Organic acids	Organic acids
mws0213	Ribitol	Others	Saccharides
mws0214	D-Sorbitol	Others	Saccharides
mws0219	L-Aspartic Acid*	Amino acids and derivatives	Amino acids and derivatives
mws0227	L-Leucine*	Amino acids and derivatives	Amino acids and derivatives
mws0242	Suberic Acid	Organic acids	Organic acids
mws0254	L-Histidine	Amino acids and derivatives	Amino acids and derivatives
mws0262	L-Tartaric acid	Organic acids	Organic acids
mws0369	Arachidonic Acid	Lipids	Free fatty acids
mws0438	L-Arabitol	Others	Saccharides
mws0470	Methylmalonic acid*	Organic acids	Organic acids
mws0473	2-Methylsuccinic acid*	Organic acids	Organic acids
mws0567	4-Guanidinobutyric acid	Organic acids	Organic acids
mws0572	5-Methylcytosine	Nucleotides and derivatives	Nucleotides and derivatives
mws0597	5-Hydroxyindole-3-acetic acid	Alkaloids	Plumerane
mws0609	Guanosine 3',5'-cyclic monophosphate	Nucleotides and derivatives	Nucleotides and derivatives
mws0629	L-Aspartyl-L-Phenylalanine	Amino acids and derivatives	Amino acids and derivatives
mws0671	L-Homoserine	Amino acids and derivatives	Amino acids and derivatives

mws0823	3-Methyl-2-Oxobutanoic acid	Organic acids	Organic acids
mws0847	1-Methyladenine	Nucleotides and derivatives	Nucleotides and derivatives
mws0853	Sinapyl alcohol*	Phenolic acids	Phenolic acids
mws0914	3,5,7-Trihydroxyflavanone (Pinobanksin)*	Flavonoids	Flavanonols
mws0924	2-Methylglutaric acid*	Organic acids	Organic acids
mws0976	β-Pseudouridine	Nucleotides and derivatives	Nucleotides and derivatives
mws1002	Syringetin	Flavonoids	Flavonols
mws1013	Esculetin (6,7-Dihydroxycoumarin)	Lignans and Coumarins	Coumarins
mws1014	Fraxetin (7,8-Dihydroxy-6-methoxycoumarin)	Lignans and Coumarins	Coumarins
mws1015	Esculin (6,7-Dihydroxycoumarin-6-O-glucoside)*	Lignans and Coumarins	Coumarins
mws1024	p-Coumaraldehyde	Phenolic acids	Phenolic acids
mws1050	O-Acetylserine	Amino acids and derivatives	Amino acids and derivatives
mws1068	Kaempferol (3,5,7,4'-Tetrahydroxyflavone)	Flavonoids	Flavonols
mws1074	Daphnetin	Lignans and Coumarins	Coumarins
mws1078	Anthranilic Acid	Phenolic acids	Phenolic acids
mws1094	Aromadendrin (Dihydrokaempferol)	Flavonoids	Flavanonols
mws1164	D-Fructose*	Others	Saccharides
mws1189	D-Galacturonic acid*	Others	Saccharides
mws1299	Luteolin-8-C-glucoside (Orientin)*	Flavonoids	Flavones
mws1336	4-Aminobenzoic acid	Phenolic acids	Phenolic acids
mws1358	Pyrocatechol	Phenolic acids	Phenolic acids
mws1433	N-Feruloyltyramine; Moupinamide	Alkaloids	Phenolamine
mws1474	5,7-Dihydroxy-3',4',5'-trimethoxyflavone	Flavonoids	Flavones
mws1489	Stearic Acid	Lipids	Free fatty acids

MWS1852	4-Methoxybenzaldehyde	Phenolic acids	Phenolic acids
MWS1854	4-Aminophenol	Alkaloids	Phenolamine
MWS1882	Iminodiacetic acid*	Organic acids	Organic acids
MWS1933	N-(2-Methylbenzoyl)glycine	Amino acids and derivatives	Amino acids and derivatives
MWS201397	Hyp-Val	Amino acids and derivatives	Amino acids and derivatives
MWS201400	Val-Trp	Amino acids and derivatives	Amino acids and derivatives
MWS201434	Ile-His	Amino acids and derivatives	Amino acids and derivatives
MWS201437	Leu-Asp	Amino acids and derivatives	Amino acids and derivatives
MWS201442	Phe-Thr	Amino acids and derivatives	Amino acids and derivatives
MWS201445	Val-Pro	Amino acids and derivatives	Amino acids and derivatives
MWS201458	Phe-Ile	Amino acids and derivatives	Amino acids and derivatives
MWS201465	Glu-Phe	Amino acids and derivatives	Amino acids and derivatives
MWS20152	Syringaresinol	Lignans and Coumarins	Lignans
MWS20169	2,5-dihydroxy-1-methoxy-anthraquinone	Quinones	Anthraquinone
MWS20178	isofraxidin	Lignans and Coumarins	Coumarins
MWS2076	2-Aminophenol	Alkaloids	Phenolamine
mws2108	Cryptochlorogenic acid (4-O-Caffeoylquinic acid)*	Phenolic acids	Phenolic acids

mws2118	Phloretin-2'-O-glucoside (Phlorizin)	Flavonoids	Chalcones
mws2212	Caffeic acid	Phenolic acids	Phenolic acids
mws2368	Tyrosol; 4-Hydroxyphenylethanol	Phenolic acids	Phenolic acids
MWS2417	3-Hydroxymandelate	Organic acids	Organic acids
MWS2984	8-Azaguanine	Nucleotides and derivatives	Nucleotides and derivatives
MWS3136	4-Methoxysalicylic Acid	Phenolic acids	Phenolic acids
MWS3149	3-Methoxybenzoic acid	Phenolic acids	Phenolic acids
mws4002	Dopamine	Alkaloids	Phenolamine
mws4052	1-Aminocyclopropane-1-carboxylic acid*	Organic acids	Organic acids
mws4170	D-Glucose*	Others	Saccharides
mws4175	D-Glucurono-6,3-lactone	Others	Saccharides
mws4193	Homovanillic alcohol; 4-Hydroxy-3-methoxyphenethanol	Phenolic acids	Phenolic acids
MWS4296	Glycylphenylalanine	Amino acids and derivatives	Amino acids and derivatives
MWS4301	3-hydroxyphenylacetic acid	Phenolic acids	Phenolic acids
MWS4525	6-O-methylguanine	Nucleotides and derivatives	Nucleotides and derivatives
MWS5164	N,N'-Dimethylarginine;SDMA*	Amino acids and derivatives	Amino acids and derivatives
MWS5206	4-Hydroxyphenyllactic Acid*	Phenolic acids	Phenolic acids
MWS80006	9-Hydroperoxy-9Z,11E-Octadecadienoic Acid	Lipids	Free fatty acids
MWS80007	13-Hydroperoxy-9Z,11E-octadecadienoic acid*	Lipids	Free fatty acids
MWSHC20109	Coumarin	Lignans and Coumarins	Coumarins
MWSHC20155	Scopoletin (7-Hydroxy-6-methoxycoumarin)	Lignans and Coumarins	Coumarins
MWSHC20168	Methyl cumalate*	Phenolic acids	Phenolic acids
MWSHC20170	Phenylmethyl β-L-glucopyranoside	Phenolic acids	Phenolic acids

MWSHC20172	Isoscopoletin (6-Hydroxy-7-Methoxycoumarin)	Lignans and Coumarins	Coumarins
MWSHC20189	Epipinoresinol*	Lignans and Coumarins	Lignans
MWSHC2022	Glucosyringic acid	Phenolic acids	Phenolic acids
MWSHC2025	(R)-1,2,3,4-Tetrahydro-3-carboxy-2-carboline	Alkaloids	Alkaloids
MWSHC2028	(1S,3S)-1-Methyl-1,2,3,4-tetrahydro-β-carboline-3-carboxylic acid	Alkaloids	Alkaloids
MWSHC2048	Skimmianine	Alkaloids	Quinoline alkaloids
MWSHC2054	Adenine	Nucleotides and derivatives	Nucleotides and derivatives
MWSHC2097	limonin	Terpenoids	Triterpene
MWSHY0016	Luteolin-6-C-glucoside (Isoorientin)*	Flavonoids	Flavones
MWSHY0091	Obacunone	Terpenoids	Triterpene
MWSHY0124	Pinocembrin (Dihydrochrysin)	Flavonoids	Flavanones
MWSHY0190	Diosmetin-7-O-rutinoside (Diosmin)*	Flavonoids	Flavones
MWSmce025	Fraxetin-8-O-glucoside (Fraxin)	Lignans and Coumarins	Coumarins
MWSmce068	Dictamine	Alkaloids	Quinoline alkaloids
MWSmce083	Ferulic acid methyl ester	Phenolic acids	Phenolic acids
MWSmce089	p-Coumaroyltyramine	Alkaloids	Phenolamine
MWSmce177	Ethyl ferulate	Phenolic acids	Phenolic acids
MWSmce199	L-Xylose*	Others	Saccharides
MWSmce210	Chelidonic acid	Organic acids	Organic acids
MWSmce264	Hydroxytyrosol	Phenolic acids	Phenolic acids
MWSmce283	4'-Hydroxypropiophenone	Phenolic acids	Phenolic acids
MWSmce331	Azetidine-2-carboxylic acid*	Alkaloids	Alkaloids
MWSmce370	4-Hydroxyphenylacetic acid	Phenolic acids	Phenolic acids
MWSmce376	3-O-Rhamnosyl(1→2)arabinosyl-23-hydroxylup-20(29)-en-28-oic acid (Anemoside A3)	Terpenoids	Triterpene Saponin
MWSmce383	Hederacolchiside A1	Terpenoids	Triterpene Saponin

MWSmce387	3-O-Methylgallic acid*	Phenolic acids	Phenolic acids
MWSmce394	2,3,19-Trihydroxyurs-12-en-28-oic acid (Tormentic acid)	Terpenoids	Triterpene
MWSmce399	Diosgenin-3-O-glucoside (Trillin)	Steroids	Steroidal saponins
MWSmce430	Hydroxytyrosol acetate	Phenolic acids	Phenolic acids
MWSmce437	Spirost-5-en-3-yl-[6-deoxymannosyl(1→2)]glucoside (Prosapogenin A)	Steroids	Steroidal saponins
MWSmce439	3,4-Dihydrocoumarin	Lignans and Coumarins	Coumarins
MWSmce461	L-Azetidine-2-carboxylic acid*	Alkaloids	Alkaloids
MWSmce467	4-Allylcatechol	Phenolic acids	Phenolic acids
MWSmce468	2-hydroxymethyl benzoic acid	Phenolic acids	Phenolic acids
MWSmce501	Protocatechuic Acid Methyl Ester	Phenolic acids	Phenolic acids
MWSmce548	Betaine	Alkaloids	Alkaloids
MWSmce585	Methyl 3-aminopropanoate	Amino acids and derivatives	Amino acids and derivatives
MWSmce625	2,3,5,6-Tetramethylpyrazine; Ligustrazine	Alkaloids	Alkaloids
MWSmce676	D-Arabinose*	Others	Saccharides
MWSmce686	Danshensu; Salvianic Acid A	Phenolic acids	Phenolic acids
MWSmce690	Erythorbic Acid; Isoascorbic Acid	Others	Vitamin
MWSmce707	Ethyl 3-hydroxybutyrate	Organic acids	Organic acids
MWSprf018	3-Hydroxylup-20(29)-en-28-al (Betulinaldehyde)	Terpenoids	Triterpene
MWSprf074	Raddeanin A	Terpenoids	Triterpene Saponin
MWSslk031	Indirubin	Alkaloids	Plumerane
MWSslk066	3-Hydroxy-4-methoxybenzoic acid; Isovanillic Acid	Phenolic acids	Phenolic acids
MWSslk092	Usnic acid	Phenolic acids	Phenolic acids
MWSslk108	O-Acetyl-L-carnitine	Alkaloids	Alkaloids
MWSslk114	Pherocon (4-(4-Acetoxyphenyl)-2-butanone)	Others	Ketone compounds

MWSslk124	2-Benzylsuccinic Acid	Organic acids	Organic acids
MWSslk132	Hexadecanedioic acid	Lipids	Free fatty acids
MWSslk146	4'-O-Glucosylvitexin	Flavonoids	Flavones
MWSslk155	4-Hydroxy-3-methoxymandelate	Phenolic acids	Phenolic acids
MWSslk226	Benzylacetone	Others	Ketone compounds
MWStz054	2,4,6,6-Tetramethyl-3(6H)-pyridinone	Alkaloids	Pyridine alkaloids
MWStz058	7-Hydroxy-β-carboline-1-propionic acid	Alkaloids	Plumerane
MWStz063	2-Ethyl-2,6,6-trimethylpiperidin-4-one	Alkaloids	Piperidine alkaloids
MWStz083	Cyclo(D-Val-L-Pro)	Amino acids and derivatives	Amino acids and derivatives
MWStz085	Aurantiamide	Alkaloids	Alkaloids
MWStz104	3,4-Dimethoxybenzamide	Alkaloids	Phenolamine
MWStz190	Neoechinulin A	Alkaloids	Plumerane
MWStz201	β-Carboline-1-propanoic acid	Alkaloids	Plumerane
MWStz213	Canthin-6-one	Alkaloids	Plumerane
MWStz252	8-Oxyberberine	Alkaloids	Isoquinoline alkaloids
MWStz261	Cyclo(D-Leu-L-Pro)	Amino acids and derivatives	Amino acids and derivatives
MWStz294	N-Feruloyltryptamine	Alkaloids	Plumerane
NK10251888	NG,NG-Dimethyl-L-arginine*	Amino acids and derivatives	Amino acids and derivatives
NK10256198	3,5,7,2'-Tetrahydroxyflavone; Datiscetin	Flavonoids	Flavones
pma2987	Histidinol	Alkaloids	Alkaloids
pma3724	1-O-Feruloylquinic acid*	Phenolic acids	Phenolic acids
pma6298	3-Hydroxypyridine	Alkaloids	Pyridine alkaloids

pmb0037	Hordenine	Alkaloids	Phenolamine
pmb0108	Feruloyl syringic acid	Phenolic acids	Phenolic acids
pmb0382	O-Feruloyl 4-hydroxycoumarin	Lignans and Coumarins	Coumarins
pmb0475	Gallic acid-4-O-(6'''-feruloyl)sophoroside	Phenolic acids	Phenolic acids
pmb0484	Choline	Alkaloids	Alkaloids
pmb0566	Luteolin-7-O-glucoside-5-O-arabinoside	Flavonoids	Flavones
pmb0631	Orientin-6-C-arabinoside	Flavonoids	Flavones
pmb0636	Orientin-7-O-arabinoside	Flavonoids	Flavones
pmb0681	Apigenin-8-C-Arabinoside	Flavonoids	Flavones
pmb0752	3-O-Feruloylquinic acid*	Phenolic acids	Phenolic acids
pmb0782	Piperidine	Alkaloids	Piperidine alkaloids
pmb0819	3-Indoleacetonitrile	Alkaloids	Plumerane
pmb1096	Indole	Alkaloids	Plumerane
pmb1207	O-Feruloyl 3-hydroxycoumarin	Lignans and Coumarins	Coumarins
pmb2826	L-Citramalic acid	Organic acids	Organic acids
pmb2855	L-Glutamine-O-glycoside	Amino acids and derivatives	Amino acids and derivatives
pmb2857	L-Glutamic acid-O-glycoside	Amino acids and derivatives	Amino acids and derivatives
pmb2871	1-O-Gentisoyl-β-D-glucoside*	Phenolic acids	Phenolic acids
pmb2893	N-p-Coumaroylhydroxyagmatine	Alkaloids	Phenolamine
pmb3002	Chrysoeriol-7-O-rutinoside	Flavonoids	Flavones
pmb3023	Eriodictyol-8-C-glucoside*	Flavonoids	Flavanones
pmb3041	Tricin-7-O-saccharic acid	Flavonoids	Flavones
pmb3101	2-Isopropylmalic Acid	Organic acids	Organic acids

pmc0066	2'-Deoxyinosine-5'-monophosphate	Nucleotides and derivatives	Nucleotides and derivatives
pmc0281	Ribosyladenosine	Nucleotides and derivatives	Nucleotides and derivatives
pme0001	Hesperetin-7-O-neohesperidoside(Neohesperidin)*	Flavonoids	Flavanones
pme0033	Hypoxanthine	Nucleotides and derivatives	Nucleotides and derivatives
pme0137	N-Acetyl-L-Glutamine	Amino acids and derivatives	Amino acids and derivatives
pme0166	1-Methylxanthine	Nucleotides and derivatives	Nucleotides and derivatives
pme0170	N-Acetyl-L-Arginine	Amino acids and derivatives	Amino acids and derivatives
pme0243	Glutaric acid*	Organic acids	Organic acids
pme0274	6-Aminocaproic acid	Organic acids	Organic acids
pme0275	4-Oxopentanoic Acid	Organic acids	Organic acids
pme0368	Apigenin-7-O-rutinoside (Isorhoifolin)	Flavonoids	Flavones
pme0376	Naringenin (5,7,4'-Trihydroxyflavanone)*	Flavonoids	Flavanones
pme0513	Xylitol	Others	Saccharides
pme0534	Gluconic acid	Others	Saccharides
pme1109	Guanine	Nucleotides and derivatives	Nucleotides and derivatives
pme1266	3-Methylxanthine	Nucleotides and derivatives	Nucleotides and derivatives
pme1292	Homogentisic acid*	Phenolic acids	Phenolic acids
pme1383	Pyridoxine	Others	Vitamin
pme1816	Neochlorogenic acid (5-O-Caffeoylquinic acid)*	Phenolic acids	Phenolic acids
pme2065	Dihydrozeatin	Alkaloids	Alkaloids
pme2165	Orotic acid (Vitamin B13)	Others	Vitamin
pme2380	α-Ketoglutaric acid	Organic acids	Organic acids

pme2559	N-Acetyl-L-Aspartic Acid	Amino acids and derivatives	Amino acids and derivatives
pme2598	3,4-Dihydroxybenzeneacetic acid*	Phenolic acids	Phenolic acids
pme2601	3-Hydroxypropanoic acid	Organic acids	Organic acids
pme2636	Enterodiol	Lignans and Coumarins	Lignans
pme2743	N-Phenylacetylglycine	Amino acids and derivatives	Amino acids and derivatives
pme2761	4-Hydroxy-2-oxoglutaric acid	Organic acids	Organic acids
pme2827	Palmitaldehyde	Lipids	Free fatty acids
pme2828	4-Nitrophenol	Phenolic acids	Phenolic acids
pme2914	3-Hydroxy-3-methylpentane-1,5-dioic acid	Amino acids and derivatives	Amino acids and derivatives
pme2960	Naringenin chalcone; 2',4,4',6'-Tetrahydroxychalcone*	Flavonoids	Chalcones
pme3186	DL-Glyceraldehyde-3-phosphate	Organic acids	Organic acids
pme3443	Sinapinaldehyde	Phenolic acids	Phenolic acids
pme3475	Butin; 7,3',4'-Trihydroxyflavanone*	Flavonoids	Flavanones
pme3705	D-Glucoronic acid*	Others	Saccharides
pmf0138	D-Mannose*	Others	Saccharides
pmf0139	D-Galactose*	Others	Saccharides
pmf0400	Docosanoic acid (Behenic acid)	Lipids	Free fatty acids
pmn001367	Protocatechuic acid-4-O-glucoside*	Phenolic acids	Phenolic acids
pmn001378	Pinoresinol-4-O-glucoside	Lignans and Coumarins	Lignans
pmn001506	Oleanolic acid-3-O-glucoside	Terpenoids	Triterpene Saponin
pmn001553	Cimidahurinine*	Phenolic acids	Phenolic acids
pmn001591	3,19,23-Trihydroxyurs-12-en-28-oic acid (Rutundic acid)	Terpenoids	Triterpene

pmn001681	1-(4-Methoxyphenyl)-1-propanol	Phenolic acids	Phenolic acids
pmn001697	Apigenin-7-O-glucuronide	Flavonoids	Flavones
pmn001706	2,3-Dihydroxyolean-12-en-28-oic acid (2-Hydroxyoleanolic acid)	Terpenoids	Triterpene
pmn001708	2,3,23-Trihydroxyurs-12-en-28-oic acid (Asiatic acid)	Terpenoids	Triterpene
pmp000001	Hispidulin (5,7,4'-Trihydroxy-6-methoxyflavone)*	Flavonoids	Flavones
pmp000003	Nepetin (5,7,3',4'-Tetrahydroxy-6-methoxyflavone)*	Flavonoids	Flavones
pmp000236	Isovitexin-8-O-xyloside	Flavonoids	Flavones
pmp000238	Luteolin-6-C-arabinoside-7-O-glucoside	Flavonoids	Flavones
pmp000269	2,3,19-Trihydroxyolean-12-en-28-oic acid (Arjunic acid)	Terpenoids	Triterpene
pmp000284	Fraxidin (8-Hydroxy-6,7-dimethoxycoumarin)	Lignans and Coumarins	Coumarins
pmp000357	Licoisoflavanone	Flavonoids	Other Flavonoids
pmp000358	Licoagrochalcone D	Flavonoids	Chalcones
pmp000439	3-Hydroxyurs-5(6),12,18(19)-trien-28-oic acid (Uncargenin B)	Terpenoids	Triterpene
pmp000441	3-Hydroxy-11-oxours-12-en-28-oic acid (11-Keto-ursolic acid)	Terpenoids	Triterpene
pmp000515	γ-Fagarine	Alkaloids	Quinoline alkaloids
pmp000537	Corysamine	Alkaloids	Isoquinoline alkaloids
pmp000539	Epiberberine	Alkaloids	Isoquinoline alkaloids
pmp000638	8-Prenylkaempferol	Flavonoids	Flavonols
pmp000674	Phillygenin	Lignans and Coumarins	Lignans
pmp001099	Platydesmine	Alkaloids	Quinoline alkaloids
pmp001187	1-(Dihydroxyphenyl)-N2,N3-bis(4-hydroxyphenethyl)-(5-8)-dimethoxy-1,2dihydronaphthalene-2,3-dicarboxamide	Alkaloids	Phenolamine
pmp001198	6-Deoxyfagomine	Alkaloids	Piperidine alkaloids
pmp001230	Senkyunolide M	Others	Lactones
pmp001253	Cannabisin F	Alkaloids	Phenolamine

pmp001254	Grossamide	Alkaloids	Phenolamine
pmp001284	Monopalmitin	Lipids	Glycerol ester
pmp001285	Phthalic anhydride	Phenolic acids	Phenolic acids
Qabp009500	Dehydroxy Epiruscogenin-glucose-glucoside	Steroids	Steroidal saponins
Qahn004351	Magnoloside D	Phenolic acids	Phenolic acids
Qazn004689	Forsythoside I	Phenolic acids	Phenolic acids
Qmdp090208	Codamine	Alkaloids	Isoquinoline alkaloids
Qmdp090608	N-Methylcordalmine	Alkaloids	Alkaloids
Qmfp122218	2,3-Dihydroxyurs-12,18-dien-28-oic acid (Goreishic Acid I)(ISO3)	Terpenoids	Triterpene
Qmjp110216	N-[7'-(4'-Methoxyphenyl)ethyl]-2-methoxybenzamide	Alkaloids	Alkaloids
Qmjp110317	Xylopinidine	Alkaloids	Alkaloids
Qmkp093006	Picrasidine I	Alkaloids	Plumerane
Qmqp101721	Chloranthatone	Terpenoids	Sesquiterpenoids
Qmwp111501	2-Hydroxy-4-methoxy-3-(3'-methyl-2'-butenyl)-quinoline	Alkaloids	Quinoline alkaloids
Qmyp101334	JiangxiBaiyingsu I	Terpenoids	Sesquiterpenoids
Sacn003282	10-hydroxymajoroside	Phenolic acids	Phenolic acids
Sadp007533	5-formyl-2,6-dihydroxy-1,7-dimethyl-9,10-dihydrophenanthrene	Terpenoids	Ditepenoids
Saln003605	3'-O-Feruloyl Swertiamarin	Terpenoids	Monoterpenoids
Salp005796	Fraxiresinol	Lignans and Coumarins	Lignans
Sazp004372	Bursehernin	Lignans and Coumarins	Lignans
Smcp000882	N-benzoyl-2-aminoethyl-β-D-glucopyranoside	Alkaloids	Alkaloids
Smhp003736	carlinoside	Flavonoids	Flavones
Smhp004476	Isovitexin-2"-O-xyloside*	Flavonoids	Flavones
Smln011495	Isopimaric acid	Terpenoids	Ditepenoids
Smpn009074	2,3,19,23-Tetrahydroxyurs-12-en-28-oic acid	Terpenoids	Triterpene

Wafn002827	Dihydroferulic acid glucoside	Phenolic acids	Phenolic acids
Wafn004792	1-O-Acetyl-Glucopyranose 6-Decanoate	Others	Saccharides
Wafp002988	1-O-Feruloyllysine	Alkaloids	Phenolamine
Wagp003516	Ptelefoliarine*	Alkaloids	Alkaloids
Wagp007374	3-Oxo-Alpha-Ionol	Others	Others
Walp005894	Clemiscosin C	Lignans and Coumarins	Coumarins
Walp006014	Clemiscosin A	Lignans and Coumarins	Coumarins
Wayn001257	N-(1-Deoxy-1-fructosyl)Leucine	Nucleotides and derivatives	Nucleotides and derivatives
Wayn002083	N-(1-Deoxy-1-fructosyl)Phenylalanine	Nucleotides and derivatives	Nucleotides and derivatives
Wbjp002566	(5-Methoxy-1,3-dimethyl-2-oxo-2,3-dihydro-1H-indol-3-yl)acetonitrile	Alkaloids	Alkaloids
Wbjp003503	1-methoxy-6-methyl-10-propan-2-yloxy-5,6,6a,7-tetrahydro-4H-dibenzo[de,g]quinoline-2,9-diol	Alkaloids	Alkaloids
Wbkp006892	Canthin-4-one	Alkaloids	Alkaloids
Wbmn002185	6-Hydroxy-4-(4-hydroxy-3-methoxyphenyl)-7-methoxy-naphtho[2,3-c]furan-1,3-dione-1	Lignans and Coumarins	Lignans
Wbmn003185	Kobusone	Terpenoids	Sesquiterpenoids
Wbmn003411	9-hydroxysesamin	Lignans and Coumarins	Lignans
Wbmn006600	3,4-Dihydro-4-(4-hydroxy-3-methoxyphenyl)-3-(hydroxymethyl)-6,7-dimethoxy-(3R,4S)-2-naphthalenecarboxaldehyde	Lignans and Coumarins	Lignans
Wbmn009702	2,3,23-Trihydroxyurs-12-en-28-oic acid	Terpenoids	Triterpene
Wbmn010123	Cannabifolin F	Terpenoids	Triterpene
Wbmn010483	Cannabifolin D	Terpenoids	Triterpene
Wbmp001950	1-[(4-hydroxyphenyl)methyl]-6-methoxy-2-methyl-3,4-dihydro-1h-isoquinolin-7-ol	Alkaloids	Isoquinoline alkaloids
Wbmp003302	Frambinone	Others	Ketone compounds
Wbsn013678	serratenediol diacetate	Terpenoids	Triterpene
Wbtn002952	1-(β-D-Glucopyranosyl)-3-ethyl-4-methyl-1H-pyrrole-2,5-dione	Others	Ketone compounds
Wbtn006715	1,2,4,5,8-pentahydroxy-6-methylanthracene-9,10-dione	Flavonoids	Other Flavonoids

Wbtn006810	6,7-dihydroxy-1,3-dimethoxyxanthen-9-one*	Flavonoids	Other Flavonoids
Wbtn007183	methyl 4,8-dihydroxy-6-methoxy-9-oxo-9H-xanthene-3-carboxylate	Flavonoids	Other Flavonoids
Wbtn007675	Calyxanthone	Flavonoids	Other Flavonoids
Wbtp006564	(2s)-4,8,10-trihydroxy-2-methoxy-1h,2h-furo[3,2-a]xanthen-11-one*	Flavonoids	Other Flavonoids
Wbtp007427	7,10-dihydroxy-12-methoxy-2,2-dimethyl-1,11-dioxatetracen-6-one	Others	Ketone compounds
Wcfn003299	(2S)-3,3-bis-(4-hydroxy-3-methoxyphenyl)-propane-1,2-diol	Others	Others
Wcfn004564	E-Stilstatin 3	Others	Others
Wcgn012055	Araucarolone	Terpenoids	Ditepenoids
Wchn005575	1-(4'-Hydroxy-3'-methoxyphenyl)-2-[4"-(3-hydroxypropyl)-2",6"-dimethoxyphenyl]-propane-1,3-Diol	Others	Others
Wcsn010524	(8Z,11Z)-8,11-Heptadecadienoic acid	Others	Others
Wcsn011122	(5Z,10E,14S)-14-hydroxy-2,6,10-trimethylpentadeca-5,10-dien-4-one	Others	Others
Wcsp001876	haemanthamine	Alkaloids	Alkaloids
Wdhp002535	(1r)-1-(4-hydroxyphenyl)-7-methoxy-1,2,3,4-tetrahydroisoquinolin-8-ol	Alkaloids	Isoquinoline alkaloids
Wdhp006295	N-p-Coumaroyl tyramin	Alkaloids	Isoquinoline alkaloids
Wdrp005579	6-methyl-2h,7h,8h-[1,3]dioxolo[4,5-g]isoquinolin-4-one	Alkaloids	Isoquinoline alkaloids
Wdsp003369	(1s)-7-methoxy-1-[(4-methoxyphenyl)methyl]-2-methyl-3,4-dihydro-1h-isoquinolin-6-ol	Alkaloids	Isoquinoline alkaloids
Wdsp003847	3,4,11-trimethoxy-13-methyl-7,8,12b,13-tetrahydro-5h-6-azatetraphen-10-ol	Alkaloids	Isoquinoline alkaloids
Wdsp004778	1,2,9,10-tetramethoxy-6-methyl-6H-dibenzo[de,g]quinoline	Alkaloids	Aporphine alkaloids
Wdyp002282	(1r)-1-[(4-methoxyphenyl)methyl]-1,2,3,4-tetrahydroisoquinoline-6,7-diol	Alkaloids	Isoquinoline alkaloids
Wdyp002417	1-[(4-hydroxyphenyl)methyl]-7-methoxy-2-methyl-3,4-dihydro-1h-isoquinolin-8-ol	Alkaloids	Isoquinoline alkaloids
Wdyp003530	10-(hydroxymethyl)-3,4-dimethoxy-7,8,12b,13-tetrahydro-5h-6-azatetraphen-11-ol	Alkaloids	Isoquinoline alkaloids
Wdyp004158	4,10,11-trimethoxy-13-methyl-7,8,12b,13-tetrahydro-5h-6-azatetraphen-3-ol	Alkaloids	Isoquinoline alkaloids
Wdyp004692	6,7-dihydro-4H-[1,3]dioxolo[4',5':7,8]isoquinolino[3,2-a][1,3]dioxolo[4,5-g]isoquinoline	Alkaloids	Isoquinoline alkaloids
Wdzp004802	Thalictricavine	Alkaloids	Isoquinoline alkaloids
Wmfp003458	Cyclanoline	Alkaloids	Isoquinoline alkaloids

Wmhp000055	2,5-Dimethoxybenzoquinone*	Quinones	Quinones
Wmmn002441	(E)-Linalool-1-oic acid	Lipids	Free fatty acids
Xmgp006913	2,4,2',4'-tetrahydroxy-3'-prenylchalcone	Flavonoids	Chalcones
Yacn003968	Cantleyine	Alkaloids	Alkaloids
Yaln005580	23-Hydroxytoonacilide	Terpenoids	Triterpene
Yaln007258	2-Hydroxyseneganolide	Terpenoids	Triterpene
Yalp003517	30-Hydroxyfraxinellone	Terpenoids	Sesquiterpenoids
Yamp001634	dehydroconiferyl alcohol	Others	Others
Yamp001702	1-O-Acetyl-N-desmethylrocaglamide	Flavonoids	Other Flavonoids
Yamp005633	10-((4-((3,5-dihydroxy-4-((3,4,5-trihydroxy-6-(hydroxymethyl)tetrahydro-2H-pyran-2-yl)oxy)tetrahydro-2H-pyran-2-yl)oxy)-3,5-dihydroxy-6-methyltetrahydro-2H-pyran-2-yl)oxy)-9-(hydroxymethyl)-2,2,6a,6b,9,12a-hexamethyl-1,3,4,5,6,6a,6b,7,8,8a,9,10,11,12,12a,12b,13,14b-octadecahydropicene-4a(2H)-carboxylic acid	Terpenoids	Triterpene Saponin
Yamp008350	hederagenin-3-O-β-D-glucopyranoside-4	Terpenoids	Triterpene Saponin
Ymjm000099	3-Oxooleana-11,13(18)-dien-28-oic acid	Terpenoids	Triterpene
Ymjm000140	3-O-Caffeoylquinic acid methyl ester*	Phenolic acids	Phenolic acids
Ymmg000006	Rugosic acid A	Others	Others
Yshs000048	6-Hydroxy-lupane-20(29)-en-3-one-28-oic acid	Terpenoids	Triterpene
Zabp002798	pseudocodeine	Alkaloids	Alkaloids
Zabp003633	berberrabine	Alkaloids	Alkaloids
Zahn005590	(7'S,8R,8'R)-3,5'-dimethoxy-3',4,9'-trihydroxy-7',9-epoxy-8,8'-lignan	Lignans and Coumarins	Lignans
Zahp003428	Puberulumine I	Alkaloids	Alkaloids
Zahp003631	3-(3-hydroxy-4-methylphenyl)-N-isobutylpropanamide	Alkaloids	Alkaloids
Zalp003488	4-O-Caffeoylquinic acid methyl ester*	Phenolic acids	Phenolic acids
Zalp003512	methyl 5-caffeoylquinate*	Phenolic acids	Phenolic acids

Zamn00402	sorbose*	Others	Saccharides
Zamn006847	glycoric acid	Others	Others
Zasp009163	Mudanpinoic acid A	Terpenoids	Triterpene
Zaxn007230	Isopimaradieneone*	Terpenoids	Ditepenoids
Zaxn007436	Pimaradienone*	Terpenoids	Ditepenoids
Zazp002547	cyclo-(Gly-Phe)	Amino acids and derivatives	Amino acids and derivatives
Zbdn007423	6,7-Dimethoxy-4-chromanone	Flavonoids	Other Flavonoids
Zbdp002392	Piperonylic acid	Organic acids	Organic acids
Zbjp002681	1,7-bis(4-hydroxy-3-methoxyphenyl)hept-1-ene-3-ol	Phenolic acids	Phenolic acids
Zbqn008291	3,13,15-Trihydroxyoleanane-12-one	Terpenoids	Triterpene
Zbsp004940	6-Hydroxy-7-methoxycoumarin	Lignans and Coumarins	Coumarins
Zbsp005214	Evolitrine	Alkaloids	Alkaloids
Zbzp010481	1-(3,5-Dihydroxyphenyl)pentadecan-1-one	Quinones	Quinones
Zbzp012689	methyl-8,11,14-heptadecatrienoate	Lipids	Free fatty acids
Zjbp081713	3-Hydroxyspirost-6-one-3-O-rhamnosyl-(1→2)-glucoside	Steroids	Steroidal saponins
zjbp110808	6-hydroxy-2-[2-(3-methoxy-4-hydroxyphenyl)ethyl]chromone	Others	Chromone
zjbp110819	6,7-dimethoxy-2-[2-phenylethyl]chromone	Others	Chromone
zjbp111001	5-hydroxy-6,7-dimethoxy-2-[2-(4'-methoxyphenyl)ethyl]chromone	Others	Chromone
zjbp120812	Scutebarbolide C	Terpenoids	Ditepenoids
Zjhp082627	iso-Yamogenin-Glc-Glc	Steroids	Steroidal saponins
Zjlp100907	Hederagenin-α-L-ara	Terpenoids	Triterpene Saponin
Zjlp100920	Hederagenin-β-D-glcA(1→3)-α-L-ara	Terpenoids	Triterpene Saponin
Zjmp102710	3-Hydroxy-29(or30)-al-olean-12-en-28-oic acid	Terpenoids	Triterpene
Zjmp102719	3-O-Glucosyl(1→2)arabinosyl-23-hydroxyolean-12-en-28-oic acid (Cauloside C; Akeboside Std)	Terpenoids	Triterpene Saponin

Zjmp102729	Collinsonidin	Terpenoids	Triterpene Saponin
Zjmp102732	patrinia-BII	Terpenoids	Triterpene Saponin
zjmp120216	11-Hydroxy-3-oxours-12-en-28-oic acid	Terpenoids	Triterpene
Zjzp091614	Zizyphus saponin I	Terpenoids	Triterpene Saponin
Zmbn004194	(R)-2-hydroxy-2-isobutylsuccinic acid	Others	Others
Zmcn007335	Eleutheroside I	Lignans and Coumarins	Lignans
Zmcn007871	Dipsacussaponin B	Terpenoids	Triterpene Saponin
Zmcn008856	Udosaponin A methyl ester	Terpenoids	Triterpene Saponin
Zmcn009787	23-Hydroxy-3-oxoolean-12-en-28-oic acid (Hederagonic acid)	Terpenoids	Triterpene
Zmcp007293	Dipsacoside B	Terpenoids	Triterpene Saponin
Zmcp007522	Oleanolic acid-3-O-arabinoside-28-O-glucoside(1-6)glucoside	Terpenoids	Triterpene Saponin
Zmcp010827	Spirost-5-en-3-ol (Diosgenin)	Steroids	Steroid
Zmcp102205	Spathulenol	Terpenoids	Sesquiterpenoids
Zmcp102717	Rotundifolioside D	Terpenoids	Triterpene Saponin
Zmcp107754	3-O-Rhamnosyl(1→2)arabinosyl-23-hydroxyolean-12-en-28-oic acid (Akeboside Stc;Kalopanaxsaponin A;Sapindoside A)	Terpenoids	Triterpene Saponin
Zmdn001863	2-(7-Dihydroxyl)-benzofuranyl-ferulic acid	Phenolic acids	Phenolic acids
Zmdn005358	Vladinol D	Lignans and Coumarins	Lignans
Zmdn007764	isoemodin	Quinones	Anthraquinone
Zmdn011161	Chrysophanol-9-anthrone	Quinones	Anthraquinone
Zmdp000292	Arginine methyl ester*	Amino acids and derivatives	Amino acids and derivatives
Zmgn000503	2,3-Dihydroxy-3-Methylbutanoic Acid	Organic acids	Organic acids
Zmgn005057	9,10-Epoxyoctadecanoic Acid	Lipids	Free fatty acids
Zmhp003322	Isovitexin-2''-O-(6'''-p-coumaroyl)glucoside	Flavonoids	Flavones

Zmjn001813	Pimelic acid*	Organic acids	Organic acids
Zmjp000182	N-Monomethyl-L-arginine*	Amino acids and derivatives	Amino acids and derivatives
Zmjp003179	Orientin-2"-O-xyloside	Flavonoids	Flavones
Zmjp003463	Vitexin-2"-O-xyloside*	Flavonoids	Flavones
Zmjp004928	Vanillyl-O-Vitexin	Flavonoids	Flavones
Zmln000899	Syringaldehyde-4-O-glucoside	Phenolic acids	Phenolic acids
Zmmp001429	Glycyl-L-leucine	Amino acids and derivatives	Amino acids and derivatives
Zmmp003920	H-ILE-TRP-OH	Amino acids and derivatives	Amino acids and derivatives
Zmpn002553	Cichoriin*	Lignans and Coumarins	Coumarins
Zmsp001834	2,6-Dimethoxy-1,4-benzoquinone*	Quinones	Quinones
Zmsp00487	Allantoin	Organic acids	Organic acids
Zmtn001464	4,8-Dihydroxyquinoline-2-carboxylic acid	Organic acids	Organic acids
Zmtn001661	Vanilloloside	Phenolic acids	Phenolic acids
Zmtp102902	α-Hydrojuglone glucoside	Quinones	Quinones
Zmwp003002	protosinomenine	Alkaloids	Alkaloids
Zmwp006493	8-Hydroxylindestenolide	Others	Lactones
Zmxp005324	Dihydrodehydrodiconiferyl alcohol	Lignans and Coumarins	Lignans
Zmyn000108	D-Saccharic acid	Others	Saccharides
Zmyn000268	2,3-Dihydroxypropanal	Others	Saccharides
Zmyn002323	2-Hydroxyphenylacetic acid	Organic acids	Organic acids
Zmyn005026	16-Methylheptadecanoic acid	Lipids	Free fatty acids
Zmyn005252	3-Hydroxy-palmitic acid methyl ester	Lipids	Free fatty acids

Zmyn006385	Avenanthramide G	Alkaloids	Phenolamine
Zmyn007277	Avenanthramide D	Alkaloids	Phenolamine
Zmzn000079	D-Erythrose-4-phosphate	Others	Saccharides
Zmzn003953	13(s)-hydroperoxy-(9z,11e,15z)-octadecatrienoic acid	Lipids	Free fatty acids
Zmzp006857	Tricin-4'-O-[β-guaiacyl-(9''-O-acetyl)glycerol]ether	Flavonoids	Flavones
Zmzp006906	Tricin-4'-O-[β-guaiacyl-(9''-O-p-coumaroyl)glycerol]ether	Flavonoids	Flavones
